# Correction: Metabolomics based predictive biomarker model of ARDS: A systemic measure of clinical hypoxemia

**DOI:** 10.1371/journal.pone.0193474

**Published:** 2018-02-21

**Authors:** The PLOS ONE Staff

## Notice of Republication

This article was republished on November 20, 2017 to correct errors in the author list that were introduced during the production process. The publisher apologizes for the errors. Please download this article again to view the correct version. The originally published, uncorrected article, and the republished, corrected article are provided here for reference.

## Supporting information

S1 File Originally published, uncorrected article. (PDF)

S2 File Republished, corrected article. (PDF)
