# Supplementary material for: Metabolomics based predictive biomarker model of ARDS: A systemic measure of clinical hypoxemia
Source: PLoS One. 2017 Nov 2;12(11):e0187545. doi: 10.1371/journal.pone.0187545 (PMC5667881; doi:10.1371/journal.pone.0187545)
Supplement: S1 Text — (Figure A): Representative 800 MHz 1H−13C HSQC spectrum of mBALF collected from ARDS patient depicting diseased lung-specific metabolites. (Figure B): Representative 800 MHz 1H−1H TOCSY spectrum of mBALF collected from ARDS patient depicting diseased lung-specific metabolites. (Figure C): Data normalization by Pareto scaling and log transformation. Branched chain amino acids = BCA. (Figure D): a) Two-dimensional and b) Three-dimensional score plot of principal component analysis with red color representing Mild ARDS and green as Moderate/ Severe ARDS, c) Two-dimensional and d) Three-dimensional score plot of partial least squares discriminant analysis with red color representing Mild ARDS and green as Moderate/ Severe ARDS e) values of the classification performance assessed by accuracy, R2 and Q2f) third component best classifies the model shown with asterisk. Principal component = PC, partial least squares discriminant analysis = PLS-DA. (Figure E): Variables importance in projection (VIP). (Figure F): Mean ± standard error of the nine metabolites is shown with respect to the Trimethylsilylpropanoic acid concentration (relative concentration in arbitrary unit). Trimethylsilylpropanoic acid = TSP, arbitrary unit = au, Branched chain amino acids = BCA. (Figure G): a) Volcano plot with red dot showing important metabolites. b) Significant values obtained from volcano plot. PRO = Proline, LYS/ARG = Lysine/arginine, TAU = Taurine, THR = Threonine c) statistical tool empirical Bayesian analysis of metabolites to show the discerning markers d) values obtained from empirical Bayesian analysis of metabolites. Fold change = FC, false discovery rate = FDR, empirical Bayesian analysis of metabolites = EBAM. (Figure H): a) and c) Random forest classification error with accuracy b) significant metabolites on the basis of mean decrease accuracy and d) Values of mean decrease accuracy. out of bag error = OOB error. (Table A): Classification results obtained from discriminant func [file pone.0187545.s001.pdf]

Supporting Information

Metabolomics based predictive biomarker model of ARDS: a systemic measure of clinical hypoxemia

Akhila Viswan[1,2], Chandan Singh[1], Ratan Kumar Rai[1], Afzal Azim[3*] and Neeraj Sinha[1*], Arvind Kumar Baronia[3]

[1]Centre of Biomedical Research, Lucknow, Uttar Pradesh, India

[2]Faculty of Engineering and Technology, Dr. A. P. J Abdul Kalam Technical University, Lucknow, Uttar Pradesh, India

[3]Department of Critical Care Medicine, Sanjay Gandhi Postgraduate Institute of Medical Sciences, Lucknow, Uttar Pradesh, India

Supplementary contents:

NMR spectroscopy

Statistical analysis

Data normalization

Discriminant function analysis

Principal component analysis and Partial least squares discriminant analysis

Variable importance in projection (VIP)

Discriminant function analysis

Relative peak intensity

Discriminant function analysis

Cross validation details of Partial least square discriminant analysis

Volcano plot and empirical Bayesian analysis of metabolites

Random forest

NMR spectroscopy

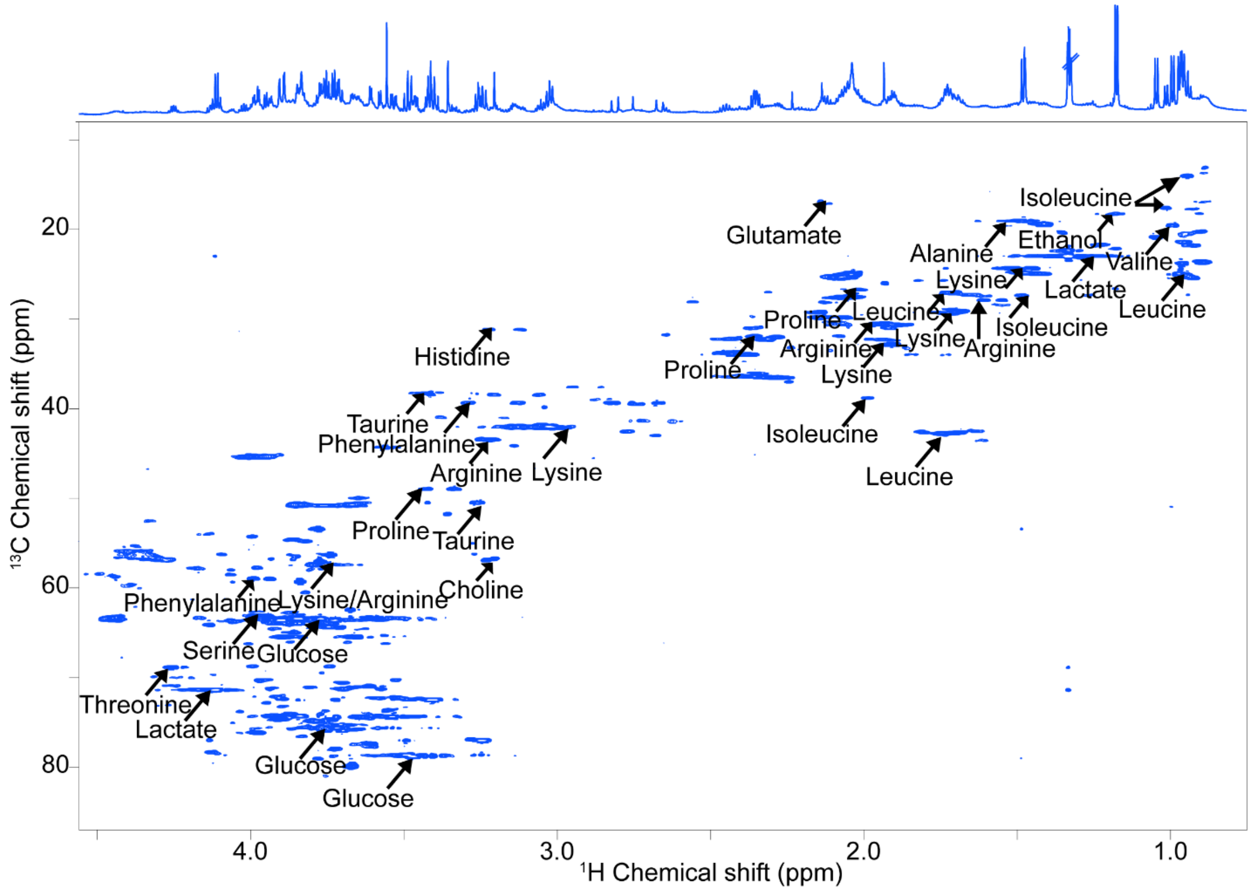

Figure A: Representative 800 MHz $^{1}H-^{13}C$ HSQC spectrum of mBALF collected from ARDS patient depicting diseased lung-specific metabolites.

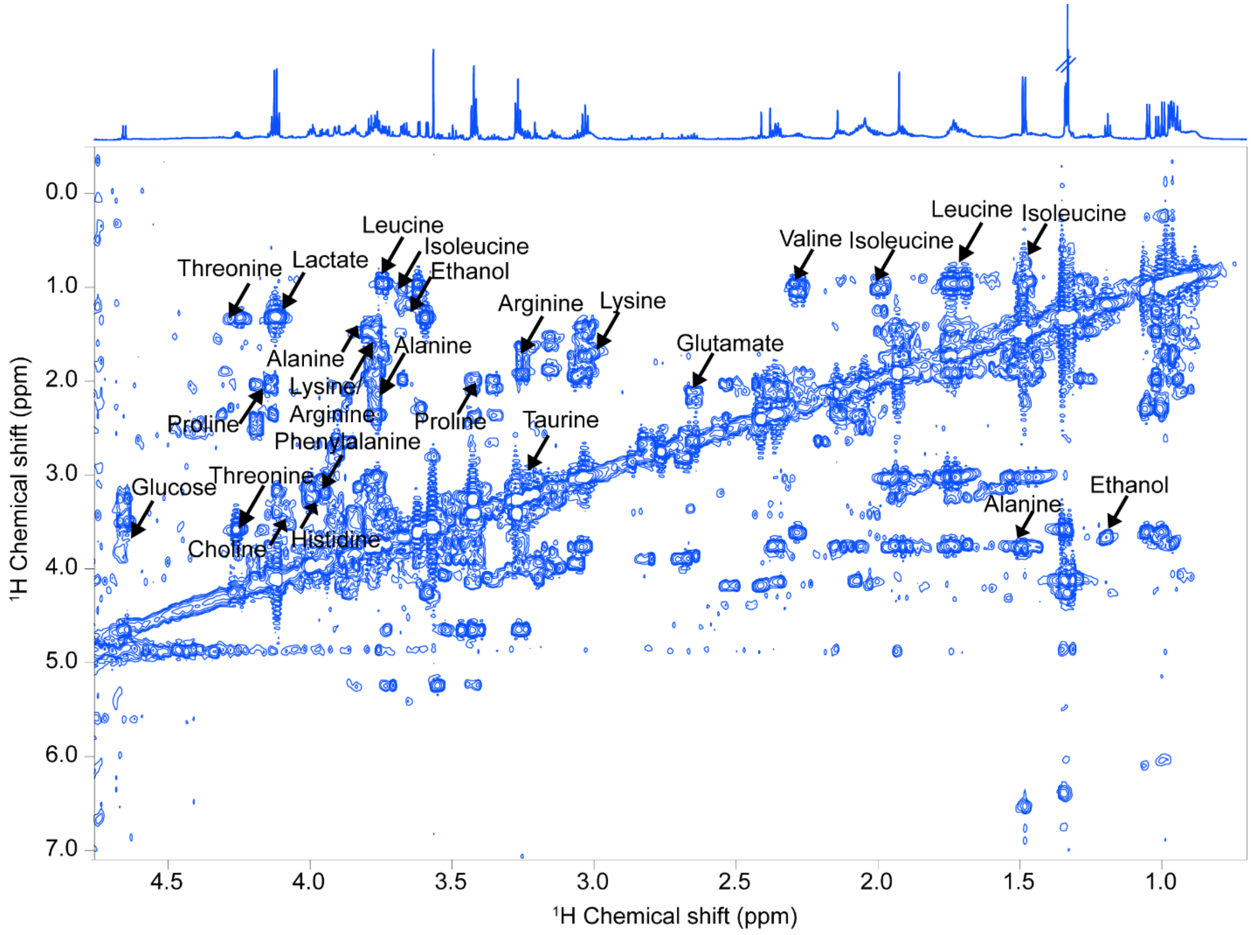

Figure B: Representative 800 MHz ^{1}H–^{1}H TOCSY spectrum of mBALF collected from ARDS patient depicting diseased lung-specific metabolites.

Statistical analysis

Among the various statistical approaches to narrow down the final putative biomarkers in the statistical modeling supervised hierarchical (agglomerative) clustering (HC) is graphically and visually represented by dendrogram and heatmap of intensities. HCA with a pictorial aid of tree dendrogram is used to explicit discrete grouping with no priori information about the data structure. In HC analysis the closely related samples are grouped into clusters based on proximities of objects and distance measure. To classify groups according to the metabolic profile we used Pearson distance measure and to minimize the sum of squares of any two clusters Wards linkage was used. Further testing the statistical significance of candidate metabolites both volcano plot and empirical Bayesian analysis of metabolites (EBAM) was conducted using Metaboanalyst. The profile of individual variable is best portrayed by volcano plot of p-value vs. log of fold change in order to culminate important metabolites. EBAM based on moderated t-statistics is a feature selection method which also outlines false discovery rate (FDR). Random forest (RF), a regression tree model based on decision trees gave out of bag (OOB) error rate which avert the need of cross validation. RF provides feature selection criteria on the basis of the impact of metabolites on the classification accuracy.

Data normalization

Before statistical analysis data was Pareto scaled and log transformed to minimize both the induced and uninduced discrepancy within the data to obtain Gaussian distribution for better interpretation of results. **(Figure C)**

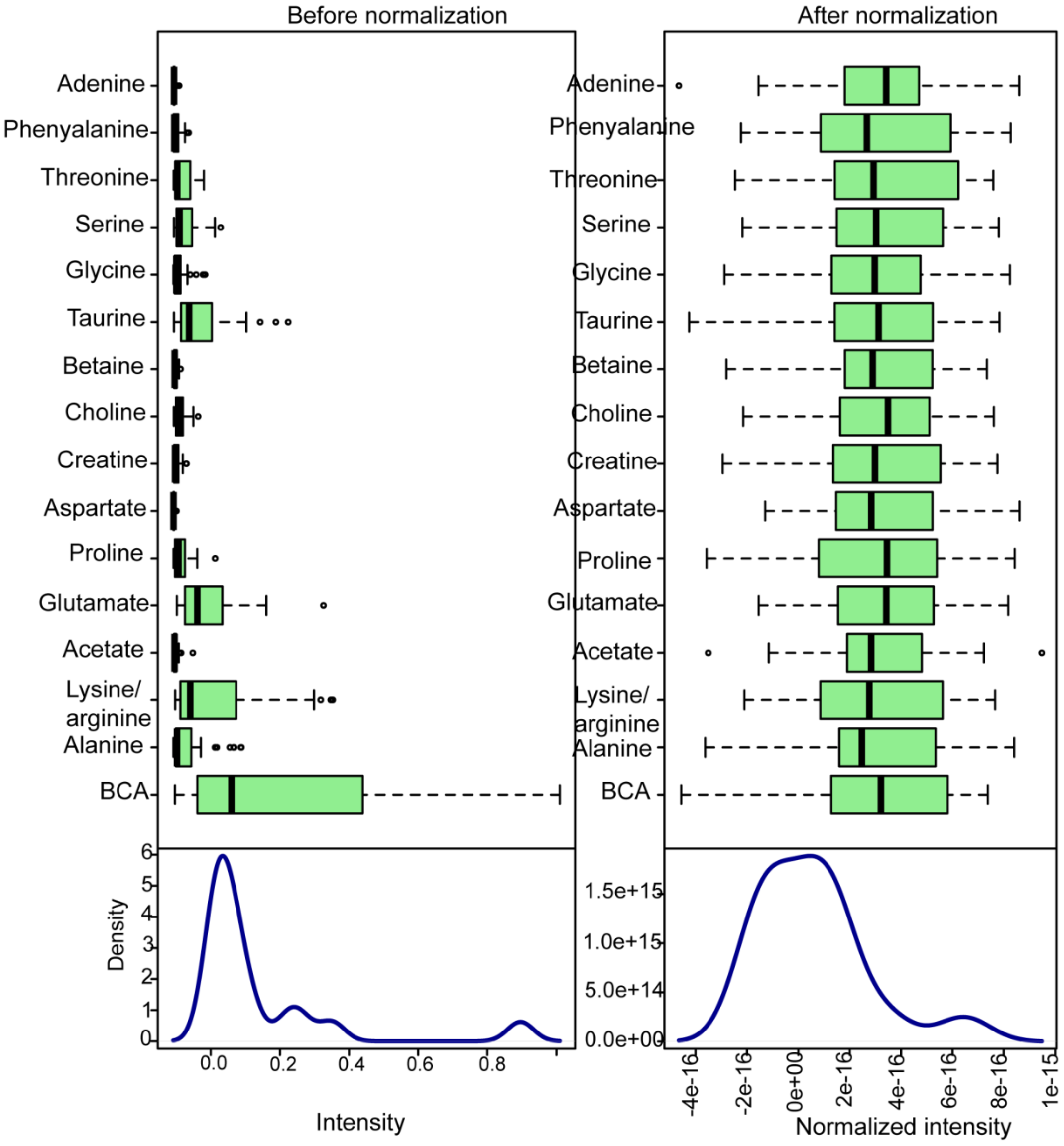

Figure C: Data normalization by Pareto scaling and log transformation.

Branched chain amino acids=BCA

Discriminant function analysis

Discriminant function analysis (DFA) was performed providing a supervised projection to minimize within group variance and maximize between group variance **(Table A)**

Table A: Classification results obtained from discriminant function analysis of 17 metabolites with prediction accuracy of 94.4%.

<table>
<tr><td colspan="3">Function</td><td>Eigen value</td><td>% of variance</td><td>Cumulative %</td><td>Canonical correlation</td></tr>
<tr><td colspan="3">1</td><td>2.7</td><td>100</td><td>100</td><td>0.854</td></tr>
<tr><td colspan="3">Test of function</td><td>Wilks lambda</td><td>Chi square</td><td>DF</td><td>Significant</td></tr>
<tr><td colspan="3">1</td><td>0.270</td><td>33.364</td><td>17</td><td>0.010</td></tr>
<tr><td colspan="3" rowspan="2">Mild ARDS/ Moderate/ Severe ARDS</td><td colspan="3">Predicted group membership</td><td rowspan="2">Total</td></tr>
<tr><td>1</td><td colspan="2">2</td></tr>
<tr><td rowspan="2">Original</td><td rowspan="2">count</td><td>1</td><td>22</td><td colspan="2">1</td><td>23</td></tr>
<tr><td>2</td><td>1</td><td colspan="2">12</td><td>13</td></tr>
<tr><td colspan="2">%</td><td>1</td><td>95.7</td><td colspan="2">4.3</td><td>100</td></tr>
<tr><td colspan="2"></td><td>2</td><td>7.7</td><td colspan="2">92.3</td><td>100</td></tr>
<tr><td colspan="7">94.4% of original grouped cases correctly classified</td></tr>
</table>

1=Moderate/ Severe ARDS, 2=Mild ARDS, DF=discriminant functions

Principal component analysis (PCA)partial least squares discriminant analysis (PLS-DA)

PCA analysis was performed to affirm grouping between the respective diseased groups of ALI and ARDS of 17 metabolites. The variance in the dataset explained by PC1 (69.4%) and PC2 (7.1%) obtained due to each variable impact on the principal component was not so striking by the 2D (score plot) and 3D representation (**Figure Da and Figure Db)**. Consequently, to improvise clustering a PLS-DA approach was ensued which yielded a better result as illustrated in (**Figure Dc and Figure Dd)**. A distinct grouping among the classes (Component1=67.3 and Component2=6.7) was lucid enough to proceed with the search of interpretative variables. The model diagnostic power and the optimal decomposition of the predicted data matrix brought by PLS-DA is evaluated from goodness of fit (R2) and cross validated R2 that is the predictive ability (Q2) values. The accuracy=0.88, R2=0.78 and Q2=0.54 was obtained from the third component (best classifier) shown with asterisk (**Figure De and Figure Df**).

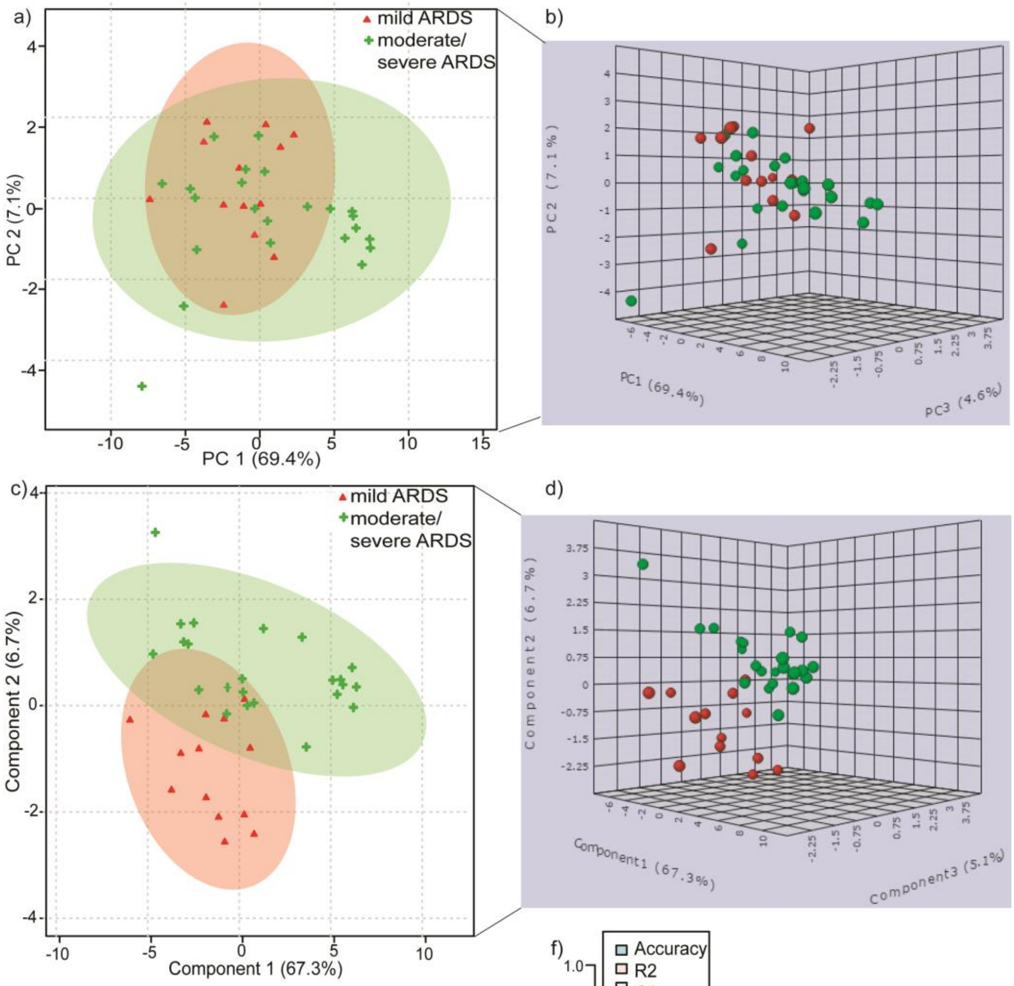

e)

PLS-DA cross validation details:

Measure	1 comps	2 comps	3 comps	4 comps	5 comps
Accuracy	0.52778	0.83333	0.88889	0.91667	0.91667
R2	0.13787	0.67465	0.78191	0.81706	0.8293
Q2	0.026415	0.42872	0.54119	0.53533	0.45643

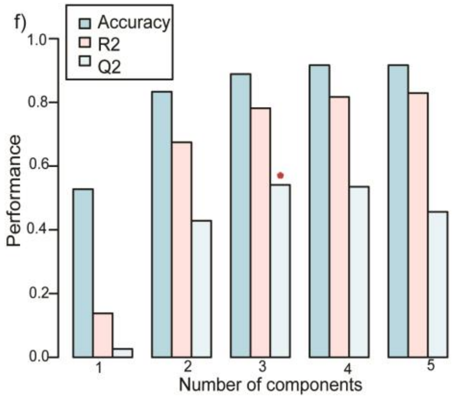

Figure D: a) Two-dimensional and b) Three-dimensional score plot of principal component analysis with red color representing Mild ARDS and green as Moderate/ Severe ARDS, c) Two-dimensional and d) Three-dimensional score plot of partial least squares discriminant analysis with red color representing Mild ARDS and green as Moderate/ Severe ARDS e) values of the classification performance assessed by accuracy, R2 and Q2f) third component best classifies the model shown with asterisk. Principal component=PC, partial least squares discriminant analysis=PLS-DA

Table B: Significant metabolites selected by T-test with a threshold p value of <0.05 analogous with Variable importance in projection values.

Biomarkers	P Values	Variable importance in projection values
Phenylalanine	0.005	1.57
Threonine	0.017	1.09
Glycine	0.005	1.121
Taurine	0.006	1.122
Proline	0.001	1.90
Lysine/ Arginine	0.004	1.38
Alanine	0.003	1.19
Branched chain amino acids	0.032	0.858
Glutamate	0.434	0.881

Variable importance in projection (VIP)

Metabolites depicted significant with a cutoff score ≥ 1**(Figure E)**

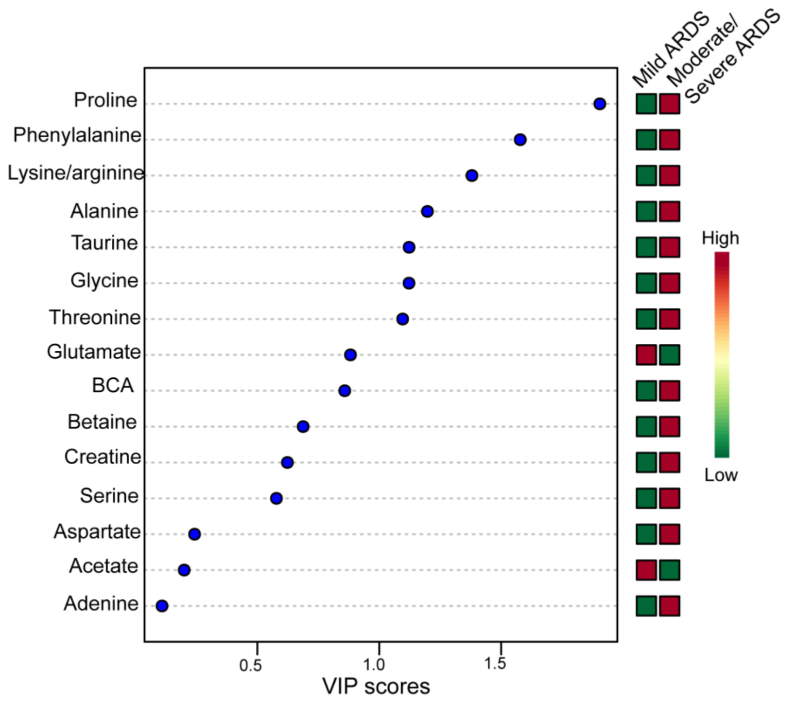

Figure E: Variables importance in projection (VIP)

Relative peak intensity

Mean ±standard error of peak intensity corresponding to 9 different metabolites is shown

(Figure F)

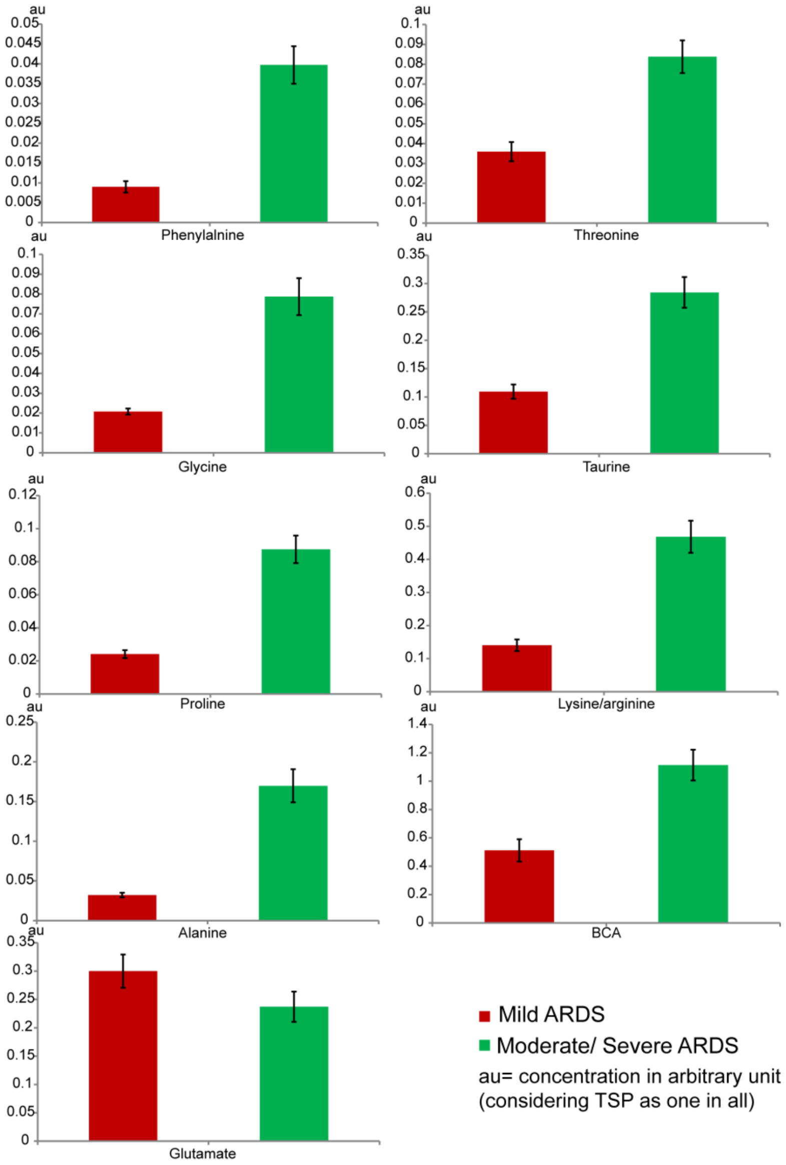

Figure F: Mean ± standard error of the nine metabolites is shown with respect to the Trimethylsilylpropanoic acid concentration (relative concentration in arbitrary unit). Trimethylsilylpropanoic acid=TSP, arbitrary unit=au, Branched chain amino acids=BCA

Discriminant function analyses (DFA)

Table C: Discriminant function analyses of nine metabolites with 88.9% correct classification.

Mild ARDS/ Moderate/ Severe ARDS			Predicted group membership		Total
			1	2	
Original	count	1	21	2	23
		2	2	11	13
%		1	91.3	8.7	100
		2	15.4	84.6	100
88.9% of original grouped cases correctly classified					

1: Moderate/ Severe ARDS, 2: Mild ARDS

Discriminant function analyses (DFA)

Table D: The classification result of stepwise discriminant function analysis to weed out 5 discriminating markers with 83.3%correct classification.

Mild ARDS/ Moderate/ Severe ARDS			Predicted group membership		Total
			1	2	
Original	count	1	21	2	23
		2	4	9	13
%		1	91.3	8.7	100
		2	30.8	69.2	100
83.3% of original grouped cases correctly classified					

1: Moderate/ Severe ARDS, 2: Mild ARDS

Cross validation details of Partial least square discriminant analysis (PLS-DA)

Table E: Partial least squares discriminant analysis cross validation details of 6 putative biomarkers with values of the classification performance assessed by accuracy, R2 and Q2and third component best classifies the model

Measure	1 component	2 component	3 component	4 component	5 component
Accuracy	0.8913	0.8913	0.91304	0.91304	0.8913
R2	0.72137	0.72628	0.72867	0.7287	0.7287
Q2	0.54367	0.5992	0.60553	0.60369	0.60343

Volcano plot and empirical Bayesian analysis of metabolites (EBAM)

Volcano plot clearly portrayed these markers on the basis of magnitude change in the predefining group with respect to their statistical significance. The scatter plot showed features with fold change (x) of 2 and t-test threshold(y) of 0.05 and both fold change and p values were log transformed **(Figure Gb)**. Important features (proline, lysine/arginine, threonine, taurine) shown with red dots had values above this threshold. The feature is attributed significant by its position that should be distant from (0, 0) **(Figure Ga)**. In conjunction with the results stated above EBAM based on moderate t-statistics was applied for much greater statistical inference which reproduced the results including glutamate **(Figure Gc)**. EBAM uses a two group mixture model for null and significant features, posteriors estimators were deduced with default delta value of 0.9. Variable is considered significant if its default delta value is equal or above 0.9. EBAM employs local false discovery rate (lfdr) as its threshold for a variable specific measure and the estimated significant values is depicted in **Figure Gd.**

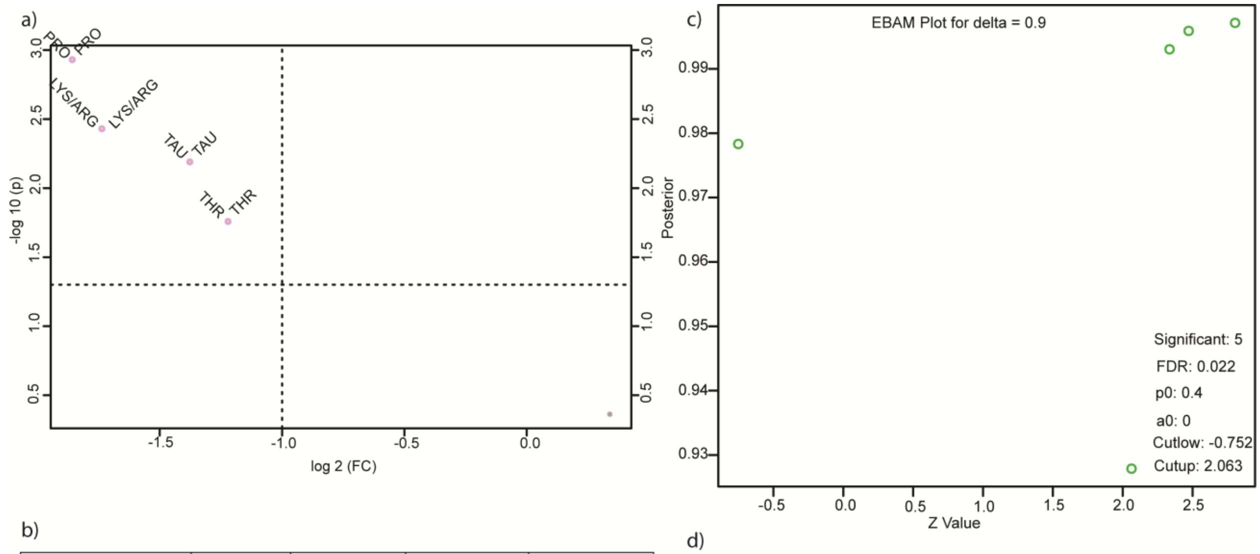

b)

Metabolites	FC	log2 (FC)	P value	-log 10 (P)
Proline	0.27592	-1.8577	.001175	2.9299
Lysine/arginine	0.30006	-1.7367	0.0037162	2.4299
Taurine	0.38498	-1.3771	0.0064598	2.1898
Threonine	0.42902	-1.2209	0.01744	1.7584

d)

Metabolites	Z value	Posterior	Local FDR
Proline	2.8046	0.99714	0.0028607
Lysine/Arginine	2.4723	0.9959	0.0041047
Taurine	2.3355	0.99304	0.0069635
Threonine	2.0633	0.92788	0.072124
Glutamate	0.75199	0.97832	0.021677

Figure G: a) Volcano plot with red dot showing important metabolites. b) Significant values obtained from volcano plot. PRO=Proline, LYS/ARG=Lysine/arginine, TAU=Taurine, THR= Threonine c) statistical tool empirical Bayesian analysis of metabolites to show the discerning markers d) values obtained from empirical Bayesian analysis of metabolites. Fold change=FC, false discovery rate=FDR, empirical Bayesian analysis of metabolites=EBAM

Random forest

A regression tree model named random forest was applied to get OOB test error estimate 13.9% with prediction accuracy 87% which is a supervised approach to rank variables. Conjointly the features have been ascribed significant on the basis of mean decrease accuracy **(Figure H)**.

a)

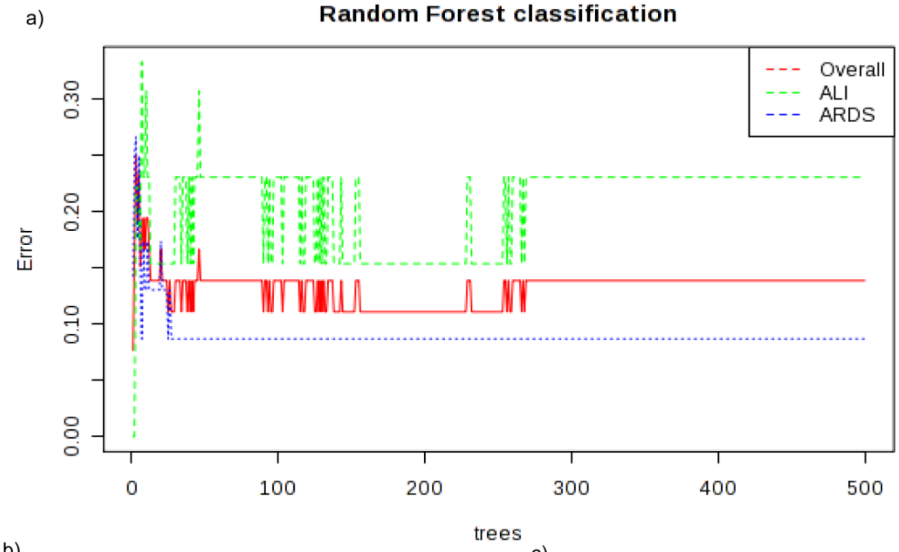

b)

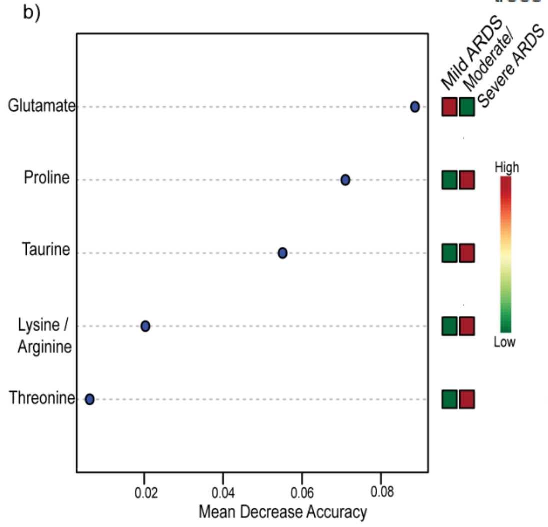

c)

OOB Error is 0.139			
	Mild ARDS	Moderate/ Severe ARDS	Classification error
Mild ARDS	11	2	0.154
Moderate/ Severe ARDS	3	20	0.13
Number of tress grown: 500<br>Number of predictors tried at each node: 7			

d)

Glutamate	0.081413
Proline	0.068464
Taurine	0.045891
Lysine/Arginine	0.031524
Threonine	0.0054401

Figure H: a) and c) Random forest classification error with accuracy b) significant metabolites on the basis of mean decrease accuracy and d) Values of mean decrease accuracy. out of bag error=OOB error
