# Supplementary material for: Correction: Metabolomics based predictive biomarker model of ARDS: A systemic measure of clinical hypoxemia
Source: PLoS One. 2018 Feb 21;13(2):e0193474. doi: 10.1371/journal.pone.0193474 (PMC5821390; doi:10.1371/journal.pone.0193474)
Supplement: S1 File — (PDF) [file pone.0193474.s001.pdf]

PLOS ONE

RESEARCH ARTICLE

Metabolomics based predictive biomarker model of ARDS: A systemic measure of clinical hypoxemia

Neeraj Sinha[1]*, Akhila Viswan[1,2], Chandan Singh[1], Ratan Kumar Rai[1], Afzal Azim[3]*, Arvind Kumar Baronia[3]

1 Centre of Biomedical Research, Lucknow, Uttar Pradesh, India, **2** Faculty of Engineering and Technology, Dr. A. P. J Abdul Kalam Technical University, Lucknow, Uttar Pradesh, India, **3** Department of Critical Care Medicine, Sanjay Gandhi Postgraduate Institute of Medical Sciences, Lucknow, Uttar Pradesh, India

* afzala@sgpgi.ac.in (AA); neeraj.sinha@cbmr.res.in (NS)

Abstract

Despite advancements in ventilator technologies, lung supportive and rescue therapies, the outcome and prognostication in acute respiratory distress syndrome (ARDS) remains incremental and ambiguous. Metabolomics is a potential insightful measure to the diagnostic approaches practiced in critical disease settings. In our study patients diagnosed with mild and moderate/severe ARDS clinically governed by hypoxemic P/F ratio between 100–300 but with indistinct molecular phenotype were discriminated employing nuclear magnetic resonance (NMR) based metabolomics of mini bronchoalveolar lavage fluid (mBALF). Resulting biomarker prototype comprising six metabolites was substantiated highlighting ARDS susceptibility/recovery. Both the groups (mild and moderate/severe ARDS) showed distinct biochemical profile based on 83.3% classification by discriminant function analysis and cross validated accuracy of 91% using partial least squares discriminant analysis as major classifier. The predictive performance of narrowed down six metabolites were found analogous with chemometrics. The proposed biomarker model consisting of six metabolites proline, lysine/arginine, taurine, threonine and glutamate were found characteristic of ARDS sub-stages with aberrant metabolism observed mainly in arginine, proline metabolism, lysine synthesis and so forth correlating to diseased metabotype. Thus NMR based metabolomics has provided new insight into ARDS sub-stages and conclusively a precise biomarker model proposed, reflecting underlying metabolic dysfunction aiding prior clinical decision making.

Check for updates

OPEN ACCESS

Citation: Sinha N, Viswan A, Singh C, Rai RK, Azim A, Baronia AK (2017) Metabolomics based predictive biomarker model of ARDS: A systemic measure of clinical hypoxemia. PLoS ONE 12(11): e0187545. https://doi.org/10.1371/journal.pone.0187545

Editor: Anirban Bhunia, Bose Institute, INDIA

Received: August 25, 2017

Accepted: October 20, 2017

Published: November 2, 2017

Copyright: © 2017 Sinha et al. This is an open access article distributed under the terms of the Creative Commons Attribution License, which permits unrestricted use, distribution, and reproduction in any medium, provided the original author and source are credited.

Data Availability Statement: All relevant data are within the paper and its Supporting Information files.

Funding: SERB, Department of Science & Technology, INDIA (EMR/2015/001758) provided financial support to NS.

Competing interests: The authors have declared that no competing interests exist.

Introduction

Acute respiratory distress syndrome (ARDS), despite many ventilator and therapeutic measures persists with a death toll of more than 40%,which has grabbed the attention of intensivists worldwide[1]. With a incidence of 58.7 cases per 100,000 population [2], efforts are needed to understand ARDS progression, aid prognostication and predict outcome with better

follow up and surveillance. It is clinically manifested by acute respiratory hypoxemia, bilateral pulmonary infiltrates and noncardiogenic pulmonary edema resulting from direct or indirect pulmonary injury[3,4]. Since ARDS first depiction in 1967 [5], several studies have been carried out to address its underlying heterogeneity, complex etiological factors and epidemiology which misleads diagnosis, risk stratification and subsequent therapeutic intervention[6]. Despite large number of rescue therapies,[7–11]the diagnostic criteria do not suffice in the management of ARDS[12–15]. The new diagnostic criteria based on Berlin definition[4] convened in 2012categorizes ARDS patient on the basis of partial pressure of oxygen to the fraction of inspired oxygen (P/F) ratio between 200–300, 100–200 and below 100 into mild, moderate and severe group respectively. But the disparity in clinical definitions so far proposed with no specific pharmacotherapy and many predisposing factors (sepsis, pneumonia, trauma, aspiration, pancreatitis)[16,17]have added to ARDS morbidity, thus necessitating symptomatic biomarkers. In continuum biomarkers of ARDS (interleukins, angiopoietin-2, and surfactant proteins) [18–20] have been explored for high risk prediction and to predict the mortality outcome. Biomarkers with predictive value, accuracy, specificity and sensitivity would permit a comprehensive evaluation of underlying predisposition to disease[21,22]. In search of biomarkers with high discriminatory ability, different biofluids have been studied extensively for better prognostication which has always served as a conventional indicator of disease pathophysiology[23,24].

The basis of further exploratory studies in ARDS relies on omics approaches by using biofluids to monitor the cellular metabolism in the diseased state. Though genomics[25] and proteomics[26]based studies have made significant progress in targeting pathological derangements but metabolomics has complemented and facilitated the contemporary understanding of dynamic, systemic and functional aspects of ARDS pathophysiology[27]. Metabolomics gives a comprehensive overview of the metabotype in response to exogenous, endogenous and environmental stimuli thus holds the novel aspect of providing readout of real time in-vivo phenotype from the top-down biochemical phenomena[28]. Many disease implicated markers of ARDS and related pulmonary diseases have been reported and probed in serum[29], exhaled breath condensate[30], undiluted pulmonary edema fluid[31], plasma[32], whole blood for gene expression analysis[33], urine[34], bronchoalveolar lavage fluid (BALF)[35–41] employing metabolomics. The simultaneous targeted and untargeted metabolite profiling, computing the inherent variance and its quantitation in response to disease or drug relies on robust, reproducible and time conducive analytical platform requiring minimal sample preparation[42]. The multiple precipitating factors of ARDS has gained notable interest among clinicians to rely on method that is unbiased, unambiguous and renders multiparametric snapshot of molecular level changes aiding both qualitative and quantitative therapeutic response to disease. Nuclear magnetic resonance (NMR) based metabolomics has emerged out as a reliable method for detecting distinct chemical signature of each metabolite and its relative perturbation in diseased state non-invasively with least sample processing thus enabling dynamic reflection of pathophysiology in biofluids[43,44]. ARDS which is radiological and clinically attributed by edema, infiltrates and inflammation of lungs requires localized epithelial lining fluid to best capture the cellular and biochemical changes[45]. A recent study has established mini Bronchoalveolar lavage fluid (mBALF)[46]from the proximal alveoli as diagnostic biofluid reflecting pulmonary metabolome for biomarker studies by NMR based metabolomics[47].Aiming to resolve the long standing bottleneck of ARDS prognostication and progression, NMR based phenotyping using mBALF though in its infancy has resulted in differentiating metabolites of acute lung injury (ALI) and ARDS with respect to controls[48]. But the explicit role of these metabolites as surrogate markers in terms of clinical utility and applicability is yet to be validated for clinical decision making and clinical trials.

The severity classification as per the new Berlin definition is based on the clinical index mainly P/F ratio which rules out some key ancillary variables of high risk profile thereby stressing more reliable and unambiguous insightful studies. Our study aims to better interpret ARDS severity classification using NMR based metabolomics and thereby set up metabolite credentials for a biomarker model aiding ARDS disease management and follow up. Our present study gives the distinct biochemical profile of mild and moderate/severe ARDS group based on endogenous metabolic profile. Differential biomarker candidates were categorized based on chemometric and pattern recognition methods from lung alveoli using NMR spectroscopy of mBALF. Comprehensive univariate and multivariate analysis resulted in the pivotal role of metabolites such as taurine, threonine, glutamate, proline, and lysine/arginine to metabolic perturbations in acute and mild stages of ARDS. Substantial role of these metabolites in ARDS intricate metabolic network was found to be governed by the dysregulated arginine and proline metabolism, lysine synthesis and degradation, taurine and hypotaurine metabolism, glycine, serine and threonine metabolism and glutamine and glutamate metabolism. The above mentioned analysis led to a putative biomarker model predictive of ARDS susceptibility or recuperation and the underlying dynamic biochemical behavior. Potential and prospects of the proposed biomarker model comprising these six metabolites is also evident from receiver operating characteristic (ROC) curve and significant pathway analysis impact greater than equal to 0.1. Thus metabolomics studies can correlate clinical endpoints with diseased specific markers that will be predictive, preventive and participatory. Correspondingly such biomarker model validated in large sample size holds application for early identification of high risk individuals providing metabolic and clinical variables of ARDS progression left unmapped.

Materials and methods

Patients

Total 36 samples were included in the study with 23 samples from patients diagnosed with moderate/severe ARDS and 13 samples taken from mild ARDS patients enrolled at Intensive Care Unit (ICU) admission of Sanjay Gandhi Post Graduate Institute of Medical Sciences (SGPGIMS). Patients included in the study group were suffering from mild and moderate/severe ARDS as stipulated by Berlin definition convened in the year 2012. The diagnostic criteria for moderate/severe ARDS[49] was based on the P/F ratio between 100–200 and $\leq$100 with bilateral opacities on chest radiograph that need not be explained by effusions or collapse, positive end-expiratory pressure($\geq$10cm H_2O) and respiratory system compliance. Similar diagnostic measures were followed for mild patients except the P/F ratio was taken between 200–300. Lung protective ventilation as per the ARDSNet protocol was practiced in the ICU. Exclusion criteria in the current study include patients with age less than 18 years, pregnancy, chronic obstructive pulmonary disease (COPD) patients, bronchial asthma, interstitial lung disease, and other chronic respiratory ailments.

Study protocol

The cross-sectional study was performed in ICU of a tertiary care medical center in SGPGIMS, Lucknow, and Centre of Biomedical Research (CBMR), Lucknow, India. The experimental protocol was approved by the SGPGIMS ethical committee and written informed consent was obtained from all subjects or their surrogate decision makers. The mBALF sample from mechanically ventilated patients was collected using the standardized non bronchoscopic "catheter in catheter" technique[50] in ICU within 24 hours of diagnosis. For mBALF suctioning, a soft suction, nontoxic, pyrogen free, graduated 16 French gauge/8 French gauge catheter

in catheter set was employed. The sample was collected in mucus trap using 10ml sterile distilled water, transferred to a collection vial following all the sterile aseptic precautions and then quenched in liquid nitrogen till further processing. The mBALF sampling and experiments were performed in accordance with the approved guidelines and regulations of the institute's ethical committee.

mBALF sampling and processing

The samples were vortexed and then centrifuged at 16000 rpm for 10min at 4˚C to remove cellular debris, bacteria and then supernatant preserved at -80˚C inside freezer for storage till NMR experiments were performed. The acquisition parameters were optimized for ^{1}H NMR mBALF spectra of mild and moderate/severe ARDS group in 800MHz NMR spectrometer (BrukerBiospin). The process of NMR spectral acquisition begins with preparation of 550μl sample including 200μl buffer to minimize the variation in pH with Trimethylsilylpropanoic acid (TSP), D_2O and 350μl BALF sample. TSP (6.53mM) was added in the buffer (0.1 M Na_2HPO_4/NaH_2PO_4, pH-7.4) for internal chemical shift reference along with 10% D_2O to provide a field frequency lock

NMR spectroscopy

NMR spectra of diseased sample were recorded at 800-MHz Bruker NMR spectrometer encompassed with a cryogenically cooled triple-resonance TCI (^{1}H, ^{13}C, ^{15}N, and ^{2}H lock) probe at 300.15K for one dimensional (1D) proton (^{1}H) NMR data acquisition of mBALF samples. All 1D NMR spectra was based on water pre saturation pulse sequence and acquired using a 90˚ flip angle, a 20ppm spectral width and a relaxation delay of 5 seconds, 32 transients were collected into 64k data points with an acquisition time (T_{aq}) of 1.99 seconds and 16 dummy scans. ^{1}H 1D spectra were referenced to the TSP signal ($\delta = 0.00$ ppm). The Free induction decays (FIDs) were multiplied by an exponential weighting function corresponding to a line broadening function of 0.3 Hz and zero filled before Fourier transformation. The acquired spectra were phased and baseline corrected and manually integrated with respect to TSP for chemical shift calibration and calculating relative resonance intensities of small molecular weight metabolites. Small metabolite resonances present in 1Dspectra were assigned and confirmed by using two dimensional (2D) NMR spectra including HSQC (Figure A in S1 Text), TOCSY (Figure B in S1 Text), COSY and Biological magnetic resonance bank (BMRB) database. For ^{1}H-^{13}C HSQC, a total of 2048 data points were collected and 24 scans were averaged for each of 826 increments covering spectral width of 16 ppm and 165 ppm in ^{1}H and ^{13}C dimension respectively. Prior to Fourier transform, zero filling up to 2k data points with forward linear prediction to 128 points and shifted sine- bell-squared apodization function were used in both t2 and t1 dimension. TOCSY spectra was acquired in phase sensitive mode and mixing time of 80 ms was set using MLEV spin lock. A total of 2k data points and 256 increments were acquired with 60 scans per increment. Prior to Fourier transform, FID was weighted in both dimension by sine-bell shaped window function and zero filled to 1k data points with 2s relaxation delay. Resonances present in ^{1}H NMR spectra were assigned to 29 small molecular weight metabolites in the diseased spectra. Among these, 17 metabolites with significant aberrance in concentration reported from our previous study[36] were manually integrated using Topspin 2.1.

Statistical analysis

In order to compute potential discriminating markers of mild and moderate/severe ARDS group, a series of univariate and multivariate analysis was performed using the software

Metaboanalyst[51] and SPSS 16.0. Data was Pareto scaled and log transformed, a pre-treatment method to obtain Gaussian distribution. Discriminant function analysis(DFA) was performed to get the accuracy of the model with 17 significant metabolites. PCA and PLS-DA analysis of these discriminating variables were performed to reduce the dimensionality of matrix and to maximize the covariance between predictor variables (metabolite intensities) and the response variables (class labels). These biomarker candidates were further validated by Independent Sample T-test at the univariate level to highlight the important variable with a threshold p value of < 0.05. For statistical inference VIP values (weighted sum of squares of PLS loadings) greater than 1 was used to specify discriminating variables. Sorted 9 metabolites were plotted in bar chart to get an overview of their mean concentration. DFA, PCA, PLS-DA were performed in succession, using the resonance intensities of these 9 metabolites to categorize the model and to project the latent variables (components) which best infer the response variables. These putative metabolites were further narrowed down to selective differential biomarkers by stepwise DFA. Using projection algorithms PCA and PLS-DA, these metabolites were checked for improved separation between the groups. Feature selection tools like supervised hierarchical clustering (HC), empirical Bayesian analysis of metabolites (EBAM), volcano plot and random forest (RF) led to the conclusive results. A detail of these statistical models is provided in the supplementary information. The Pathway analysis module was performed using Metaboanalyst, which incorporates pathway enrichment analysis and topology analysis based on KEGG database and employs novel algorithms and concepts to identify the utmost affected pathway in the diseased state. Area under the ROC curve(AUROC) was used as a benchmark to assess the diagnostic parameters (sensitivity and specificity) of candidate biomarker prototype responsible for validating the possible confounders within the stages of diseased groups.

Results

Patient characteristics

The clinical and baseline characteristics of the patients at the time of sampling are provided in Table 1 which includes demographic profile and illness severity scores like Acute Physiology and Chronic Health Evaluation-II(APACHE II) and Sequential Organ Failure Assessment (SOFA) which was taken at the time of admission. The mild and moderate/severe patients were age matched and reported with more or less similar co-morbidities with a higher total leukocyte count (TLC) and procalcitonin value in ARDS.

NMR snapshot of ARDS substages

A representative NMR spectrum showing the difference between mild and moderate/severe ARDS is depicted in Fig 1. The NMR spectrum from moderate/severe stage of ARDS are mostly dominated by resonances from amino acids, lactate, glucose, intermediates of tricarboxylic acid cycle and many other low molecular weight endogenous metabolites while the mild group is predominated by glutamate and choline resonances in the complex mixture of mBALF sample as per the Fig 1. In order to obtain a significant comparison across all subjects with interpretation of metabolic signatures predictive of disease, data were subjected to chemometric analysis. Out of the 29 metabolites assigned 18 were found to have anomaly in their intensity values as reported from our earlier study[36]. Due to the large variation exhibited by lactate resonance which hindered the possible interpretation of significant results, it was excluded from further statistical analysis. Lactate is also nonspecific and intrigued in terms of its diagnostic value as its upsurge is common phenomena associated with tissue hypoperfusion together with many etiologies[52]. So in the present study (36 disease samples) the

Table 1. Patient clinical and baseline characteristics at the time of sampling.

Characteristic	Mild ARDS	Moderate/Severe ARDS
**Number of patients**	13	23
**Male/ females**	9/4	15/8
**Age (Mean ± standard error)**	47.3 ± 3.18	45.8 ± 3.15
**Admission Sequential Organ Failure Assessment*** **(Mean±standard error)**	7.92 ± 0.48	10.78 ± 0.99
**Admission Acute Physiology and Chronic Health Evaluation-II (Mean ± standard error)**	13.23 ± 1.00	15.65 ± 1.19
**Partial pressure of oxygen/ fraction of inspired oxygen (P/F) Ratio*** **(Mean±standard error)**	234.61 ± 6.05	161.30 ± 7.00
**Length of ICU stay*** **(Mean±standard error)**	21.38± 1.59	16.61± 1.28
**Day of mechanical ventilation*** **(Mean±standard error)**	20.08± 1.58	15.08± 1.17
**Diagnosis**	Tropical infections: 4 Community acquired pneumonia: 3 Severe sepsis: 2 Severe acute pancreatitis:2 Guillain Barre syndrome: 1 Unknown etiology: 1	Tropical infections: 8 Community acquired: 5 pneumonia Severe sepsis: 4 Severe acute pancreatitis:3 Guillain Barre syndrome: 2 Unknown etiology: 1
**Co-morbidities**	Diabetes: 2 Hypertension:2 diabetes + hypertension: 5 Hypothyroidism:1Coronary artery disease: 2 Chronic kidney disease: 1	Diabetes:5 Hypertension: 4 diabetes + hypertension: 7 Hypothyroidism: 3 Coronary artery disease: 2 Chronic kidney disease: 2
**Total leukocyte count*** **(Mean ±standard error)**	12.67 ± 0.65	17.04± 0.72
**Procalcitonin*** **(Mean±standard error)**	1.41± 0.27	2.67±0.43
**Positive end expiratory pressure*** **(Mean±standard error)**	9.85± 0.42	11.04± 0.33
**Survival/ Nonsurvival***	6/7	6/17

*Statistically significant feature ($p < 0.05$)

https://doi.org/10.1371/journal.pone.0187545.t001

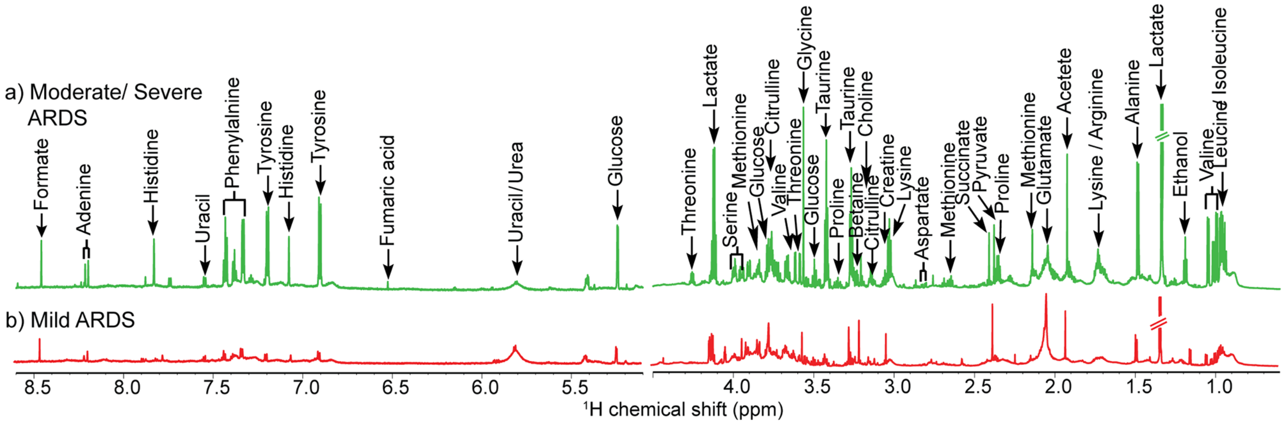

Fig 1. Representative proton nuclear magnetic resonance spectra from acute respiratory distress syndrome patients. a) Moderate/ Severe ARDS, b)Mild ARDS, proton = ^{1}H.

https://doi.org/10.1371/journal.pone.0187545.g001

17 prominent metabolites which contributed to the separation within different categories of ARDS were selected to get a snapshot of their relevance on a larger dataset. These metabolites were further substantiated for their paramount role in distinguishing 13 mild from 23 moderate/severe patients by various chemometric approaches. The study made progress with screening of 9 significant variables from 17 selected and further narrowing down to 6 potential biomarkers. Since single *a priori* statistical tool is susceptible to underlying data structure therefore successive univariate and multivariate analysis were carried out to corroborate the robustness and reproducibility of the six metabolites linked to the diseased group with their anomaly interconnected to downstream metabolic pathway.

Class separation based on significant metabolites

Although clinical disposition of ARDS and it varying heterogeneous causes and outcome adds to the uninduced biological variation, hence standardized protocols were practiced in ICU by clinicians and expertise to mitigate the amount of technical variation in sampling. All the intubated patients in ICU underwent sedation and analgesia thereby corresponding to minimal stress repercussions. Sampling has been carried out using a standardized non bronchoscopic "catheter in catheter" technique and a fixed volume of sterile distilled water was used for suctioning of tracheal aspirates by trained person. The visual interpretation of NMR generated multidimensional datasets reflects the underlying induced biological variation. This necessitates data mining and other exploratory data analysis by chemometrics with requisite data pretreatment measures[53] to remove the heteroscedasticity due to uninduced biological variation[54] mentioned above. To strengthen the classification and prediction of differential metabolites, NMR data was manually referenced to TSP and Pareto scaled[55] and log transformed[56] removing the skewed distribution because of heteroscedasticity thereby obtaining a symmetric data for further confirmatory statistical analysis.

Firstly, the DFA test classified the model with 17 metabolites as 94.4% accurate with lower Wilks lambda, significant classification as apparent from chi square test, with greater Eigen value and significant value of 0.01 (Table A in S1 Text). All the data were log transformed and Pareto scaled (Figure C in S1 Text). The PCA and PLS-DA (Figure D in S1 Text) further assured that the experimental parameters (17 metabolites) can be employed to probe for the attributes contributing to separation between mild and moderate/severe stages of ARDS. Details of PCA and PLS-DA component contributing to model significance are provided in the supplementary information. The lower values of R2 and Q2 (accuracy = 0.88, R2 = 0.78 and Q2 = 0.54) can be correlated with the separation which is being sorted within the diseased group. Amid all the 17 metabolites scrutinized, the determinant metabolites were ranked by the VIP score. High VIP score of more than 1 was used as a cutoff to include variables with discriminatory power (Table B in S1 Text). Phenylalanine, threonine, taurine, proline, lysine/arginine, alanine, glycine, branched chain amino acids (BCA) were included in the classification. In accordance with the Figure E in S1 Text all the metabolites except glutamate and acetate were notable in the mild group. The observed significant metabolites obtained in independent sample T-test with unequal variance (threshold p value <0.05 and confidence interval 95.0%) were analogous with the VIP ranked metabolites (Table B in S1 Text). These results further corroborated these set of metabolites as significant.

Chemometrics based screening of metabolites

These eight metabolites were further screened by an iterative multivariable data mining strategies. The discriminating variables were again subjected to DFA with nine metabolites including glutamate as its prominence was shown in the mild group. The mean concentration of

these nine metabolites is also assessed in arbitrary unit (au) with respect to the concentration of TSP with standard error shown as error bars (Figure F in S1 Text). An elevated level of all the metabolites is depicted in the moderate/severe group excluding glutamate which is showing an increased concentration in mild group. The PCA and PLS-DA showed improved separation with glutamate and yielded conclusive results in subsequent analysis with DFA which yielded 88.9% correct classification (Table C in S1 Text).

Six differentiating markers of ARDS disease course

Finally, stepwise DFA sorted out 6 metabolites (proline, lysine/arginine, taurine, threonine, glutamate) with 83.3% correct classification suggesting their predominance in ARDS (Table D in S1 Text). Their discriminating power is reflected both in PCA, PC 1 = 35.1% and PC 2 = 20.8% (Fig 2a) and PLS-DA Component 1 = 20.8% and Component 2 = 26% (Fig 3a). Each spectrum represents an observation (scores) and by examining the proximity of these observation due to variables contribution (loadings) under each observation within and between the cluster of Mild ARDS and Moderate/Severe ARDS the similarity and dissimilarity in metabolic profile of mild ARDS and moderate/severe ARDS can be explained. Projection of

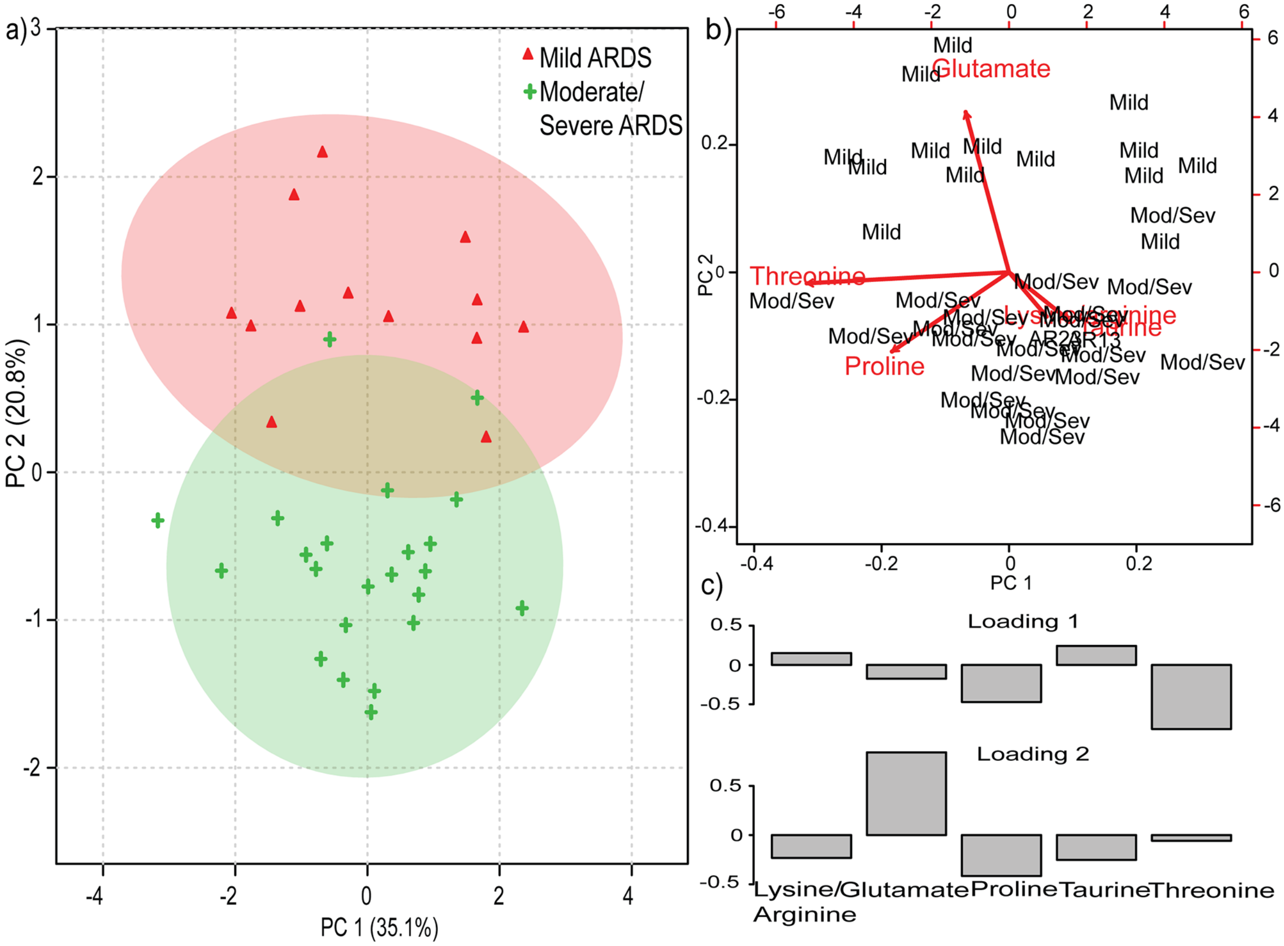

Fig 2. a) Two-dimensional score plot of principal component analysis with red colour representing mild ARDS and green as moderate/severe ARDS b) Biplot of principal component analysis c) Loading plot of principal component analysis. principal component = PC.

https://doi.org/10.1371/journal.pone.0187545.g002

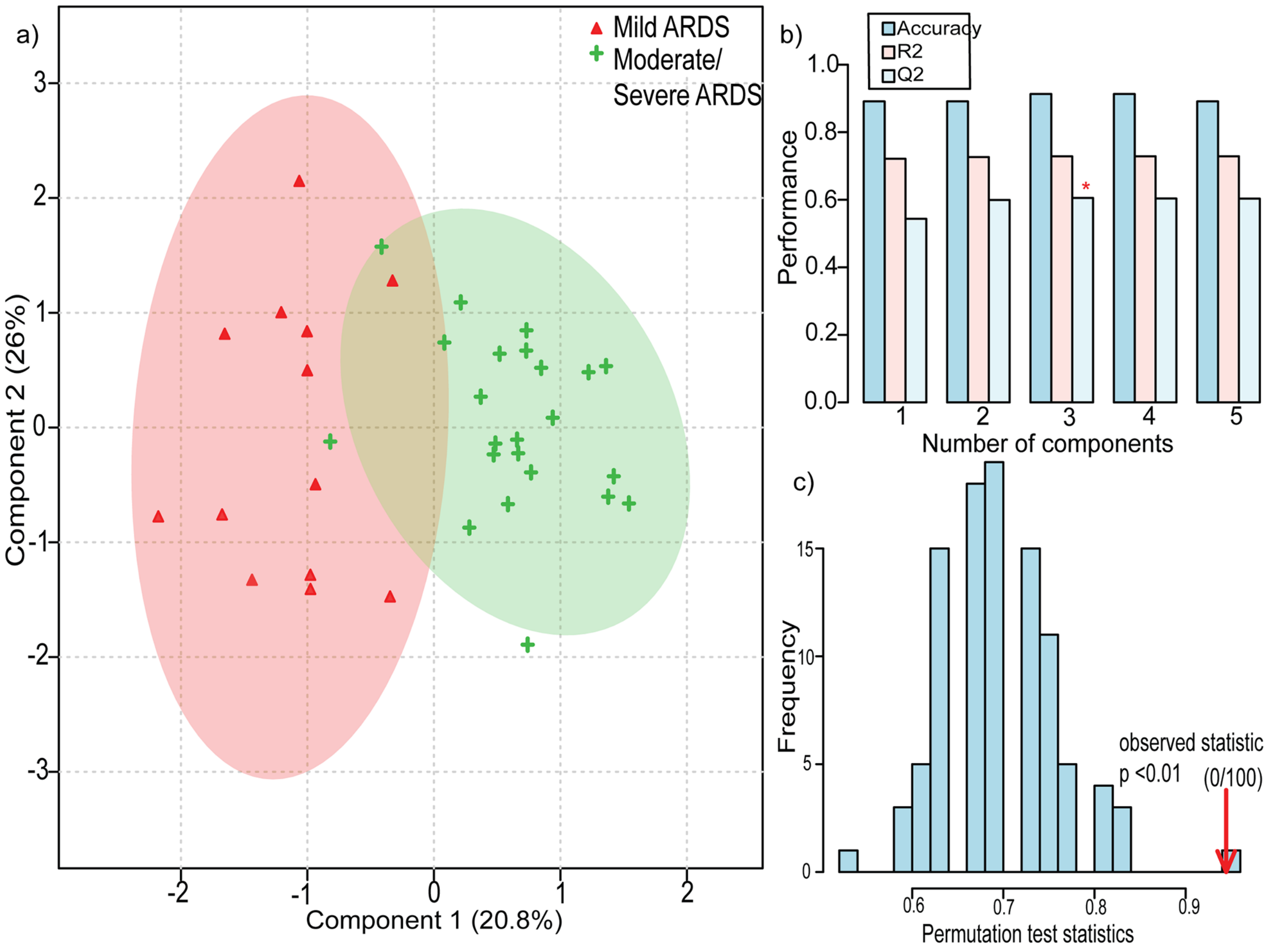

Fig 3. a) Two-dimensional score plot of partial least squares discriminant analysis with red colour representing mild ARDS and green as moderate/ severe ARDS b) third component best classifies the model shown with asterisk c) Permutation test by separation distance B/W.

https://doi.org/10.1371/journal.pone.0187545.g003

scores and loadings in two dimensional score plot of PCA and PLS-DA (Figs 2 and 3), visually revealed variance and separation between mild ARDS and moderate/severe ARDS. On the basis of PCA scores and loadings as points and arrows, a biplot (planar graph) (Fig 2b) and loading plot (Fig 2c) was plotted which provided the explanatory pattern in the data set. The six most important metabolites contributing to the 60% PCA variance were interpreted on the basis of loadings. Further biplot, a scatter plot representing an overlay of scores and loading was reduced along two principal components with arrows indicating the loadings of six metabolites, making the observation (scores) and the corresponding metabolite correlation more obvious and significant in mild ARDS and moderate/severe ARDS. Longer arrow outlines the relevance of these metabolite and distance of projection depicts its level in discriminatory group as per the Fig 2b. The Fig 2b explains the role of glutamate in mild ARDS whereas threonine, taurine, lysine/arginine, and proline contribution was found towards moderate/severe ARDS group. An improved accuracy = 0.91, predicted ($R2 = 0.72$) and expected variance ($Q2 = 0.60$) were in accordance with the above stated result using leave one out cross validation (LOOCV) (Fig 3b and Table E in S1 Text). The PLS-DA model validation by permutation test was determined by separation distance based on sum of squares between and sum of

squares within (B/W) ratio. The random class assignment prediction based on 100 permutations is plotted in histogram (Fig 3c). The p valueless than 0.01 and B/W ratio of real class assignment is calculated as shown in Fig 3c. If the real B/W ratio is not a part of the random distribution, it can be deduced that the difference between the respective groups is significant as seen in the Fig 3c.

Validation of predictive biomarker model

The selected subsets of disease specific markers (proline, lysine, arginine, threonine, taurine and glutamate) were further supported by various feature selection tools. The results were confirmed with volcano plot, EBAM (Figure G in S1 Text), random forest analysis (Figure H in S1 Text) and hierarchical clustering analysis (HCA). The details of these analyses have been provided in the supplementary information. In HCA, represented as tree dendrogram a discrete grouping is observed which signifies biological variance using the Pearson correlation and wards linkage (Fig 4a). The groups were segregated on the basis of metabolic intensities of 6 metabolites with the height of branches representing the distance between the groups. Red cluster denoting mild group and green as moderate/severe ARDS. Heat map of metabolite intensities of the 6 selective biomarkers shows correlation between the ^{1}H NMR spectral regions of selective metabolites to respective mild and moderate/severe ARDS group (Fig 4b). The predictive accuracy, specificity and sensitivity of a single biomarker is rather speculative thus an ensemble of biomarkers is sorted for more elaborate understanding of pathophysiology which governs its clinical utility. Subsequently ROC study was applied for the proposed biomarker prototype comprising proline, taurine, lysine/arginine, threonine and glutamate which came out to be 0.95 (Fig 4c).

Metabolome interaction network

The putative biomarker model comprising narrowed down metabolites pooled from biostatistics were further evaluated by generating metabolite pathway analysis highlighting the most correlated and discriminant pathway under study. The pathway analysis helped in establishing the underlying connectivity of the proposed biomarker prototype with the affected biological process based on signature specificity and metabolite impact $\geq$0.1. The most relevant and significant metabolic pathway mirroring ARDS biochemical phenomena included arginine and proline metabolism, lysine biosynthesis, lysine degradation, aminoacyl-tRNA biosynthesis, taurine and hypotaurine metabolism, glycine, serine and threonine metabolism and D-glutamine and D-glutamate metabolism, alanine, aspartate and glutamate metabolism (Fig 5a). The interaction between metabolites and the corresponding biological process involved in the diseased condition can help to probe the possible biological roles of the upregulated/ downregulated metabolites (Fig 5b). The metabolites comprising biomarker model (proline, lysine/ arginine, taurine, threonine, glutamate) were also found with significant impact in pathway analysis thereby further large sample study will help in predicting their role in susceptibility/ recovery of ARDS disease course.

A comparison study was followed up to understand the role of these biomarkers in lung physiology. The novel findings in this study arise from the fact that:(1) NMR based metabolomics can differentiate mild and moderate/severe ARDS group on the basis of discrete metabolic signature distinct from the clinical and other ancillary variables. (2) This is the first differential study executed on such sample size of ARDS employing the New Berlin definition. (3) The results have been validated independently in multivariate datasets. (4)The six selective pool of endogenous markers constituting the predictive biomarker model can reflect the

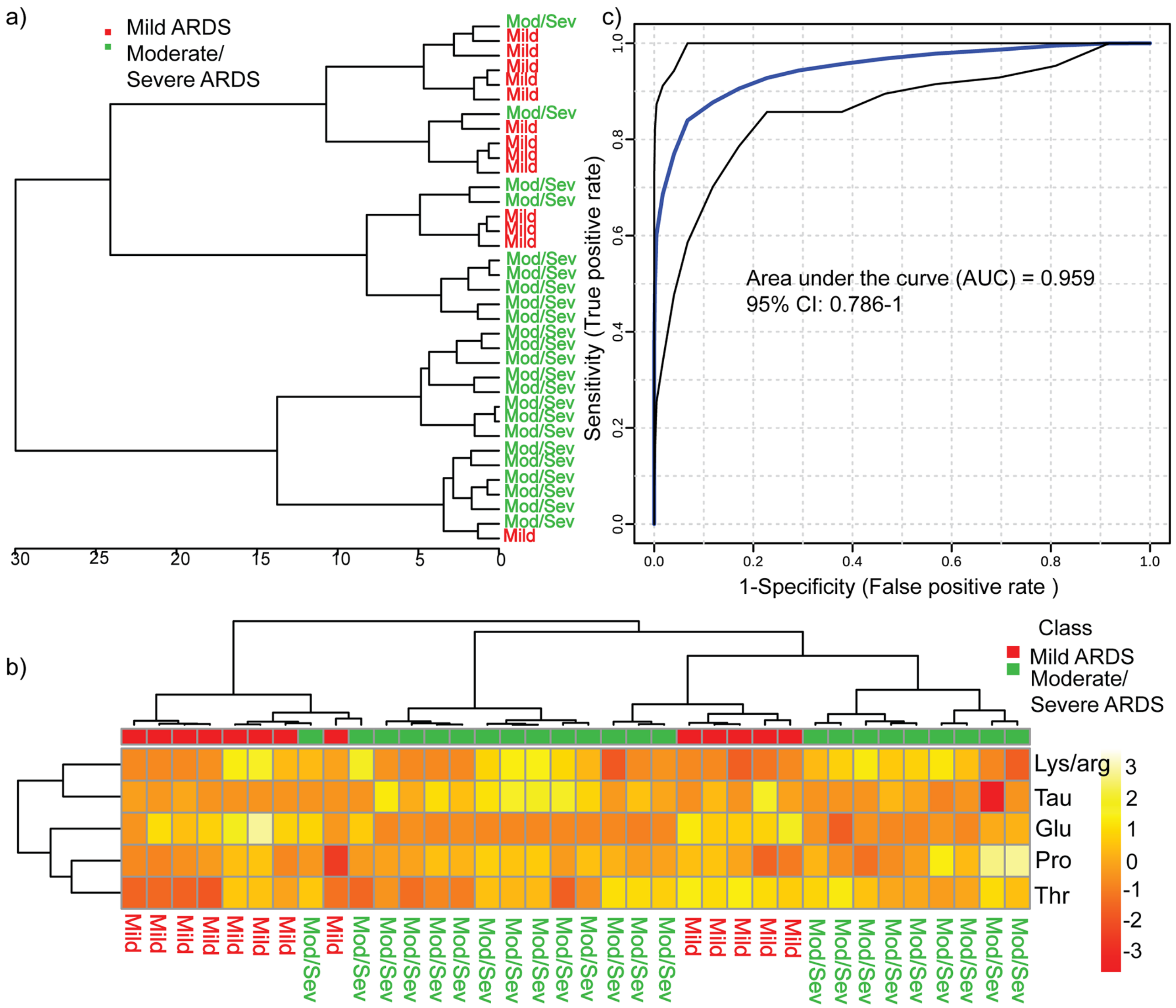

Fig 4. a) Hierarchical clustering showing clustering obtained by the discriminating potential of the selected metabolites. b) Heat map showing the relative intensity of each metabolite in different subgroups in conjunction with dendrogram. c) Area under the curve depicting the sensitivity and specificity of the proposed biomarker model with 95% accuracy.

https://doi.org/10.1371/journal.pone.0187545.g004

metabolic anomaly in ARDS substages with 95 percent accuracy defined by AUROC and the significantly associated metabolic pathway.

Discussion

Lung injury due to its many etiologies results in complex metabolic perturbation, consequently metabolites linked to the diseased state shows a surge of metabolites interlinked with the dysregulation of metabolism that can be used to address the various clinical manifestations. ARDS pathophysiology governing inflammatory reactions results in hypoxia, reactive oxygen species (ROS) production and oxidative stress which trigger oxidative attack on lysine, arginine, proline, threonine leading to carbonylated proteins. Carbonylated proteins which are oxidated proteins have found to be implicated in ARDS [57]similar to what we found elevated in our study. Glutamate was found in increased concentration in the mild group indicating its

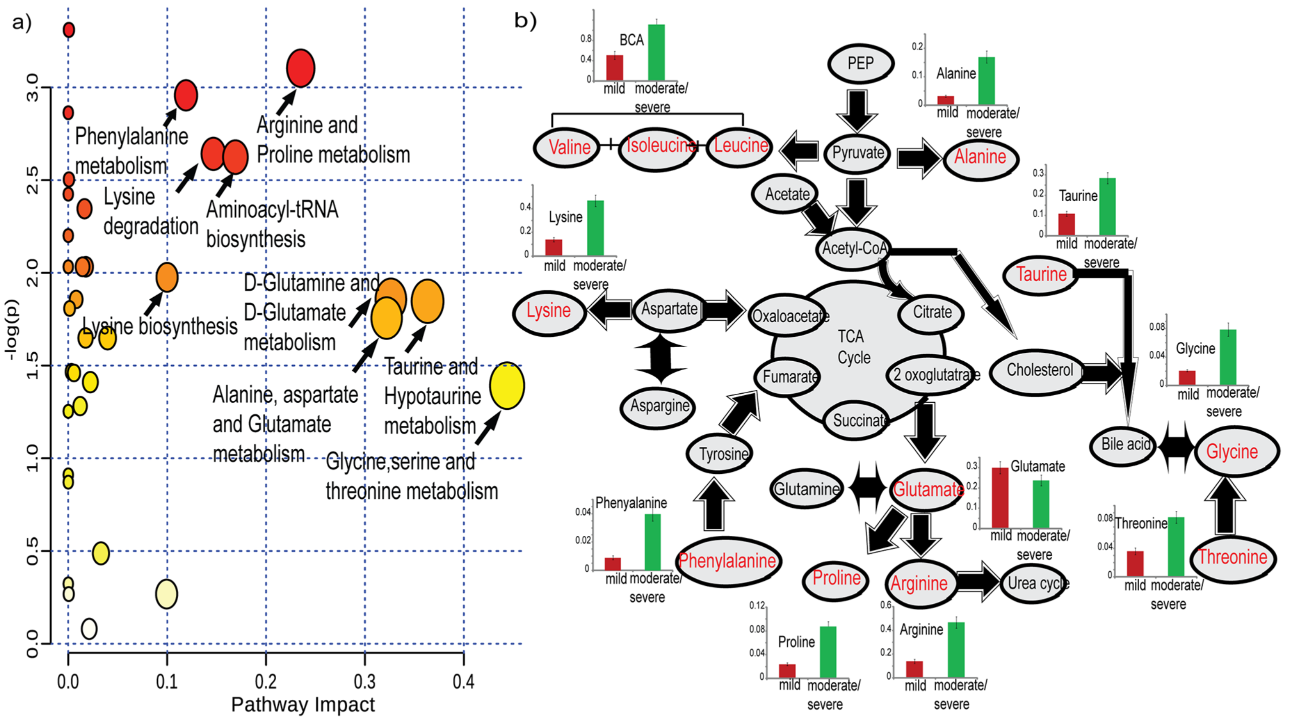

Fig 5. a) The metabolic pathways found significant in ARDS with pathway impact ≥0.1. b) The significant metabolites (in bold red color) interaction and associated biological process in ARDS substages is shown with their relative concentration in arbitrary units with respect to TSP in mild ARDS and moderate/severe ARDS.

https://doi.org/10.1371/journal.pone.0187545.g005

association with less diseased lung of ARDS. Falling in the class of excitatory neurotransmitters glutamate evidential role has been implicated in acute lung injury[58]. The role of glutamate agonist N-methyl-D-aspartate (NMDA) has been reported in the rat lungs.[59] NMDA mediates excitotoxicity through NMDA receptors present possibly in the alveolar capillary area by causing pulmonary edema[60]. Glutamate signaling can in turn evoke nitric oxide (NO) production and apoptosis attributed by caspase 3 productions. Further studies on the physiological role of glutamate signaling in lung toxicity can unravel more restorative measures for ARDS.

Macrophage and neutrophil sequestration results in the collateral release of ROS, proinflammatory cytokines hampering epithelial and endothelial functions with increased microvascular permeability due to endothelial barrier disruption. The consequent free radical production during the pathogenesis of ARDS is likely reduced by the high concentration of taurine. Taurine is an endogenous sulphur containing amino acid found in neutrophil. Taurine has already been attributed for its protective role as an antioxidant with anti-inflammatory properties. Taurine has also been found imperative in alleviating IL-2 induced lung injury by attenuating neutrophil- endothelial interaction[61]. Thus taurine escalation in ARDS is an index of rescue mechanism to check inflammation.

Another metabolite found proliferated in ARDS patients in our study was arginine. The key role of arginine has been signified in the intermediary metabolism of critically ill patients with salient effect in the periods of hypermetabolic stress[62]. Arginine is the only biosynthetic precursor of NO. Arginine upregulation can be correlated with the body defense mechanism to increase NO production to relieve pulmonary hypertension associated with hypoxia[63]. NO a

potent endothelial derived relaxing factor produced by lungs, has got pertinent roles in lung physiology and pathobiology of lung diseases[64]. Arginine can be prospective target to modulate the endothelial NO production. Lysine level was also found to be increased in ARDS and may be ascribed for its viable defensive role in ARDS. Its role has been found evident in decreasing NO production thus increasing vascular resistance as reported in neonatal pigs[65].

Another metabolite upregulated in the diseased group is threonine. Threonine a gluconeogenic amino acid is found to be associated with sepsis[66] which accounts to be a major aetiological factor of ARDS. It regulates immune responses by the production of antibodies, triggers lymphocyte proliferation with a significant role in the inhibition of apoptosis[67]. There is a paucity of literature suggesting the immunoregulatory role of threonine in the critical setting of lung physiology. Proline, a proteogenic secondary amino acid is a key precursor of collagen formation and mesenchyme in the lungs. It showed a significant increase in the ARDS group. Collagen is found to be associated with branching in airways. Airway remodelling in chronic lung diseases is reported with the production of proline due to the activity of arginase from alveolar macrophages[68]. Proline residues are vulnerable to oxidation by ROS resulting in fragmentation and apparent loss of function. Proline displays a relevant role as a stress substrate in the microenvironment of inflammation[69].

These metabolites (lysine, arginine, proline, threonine, taurine, glutamate) can be used as a possible measure to illustrate the complex pathophysiology associated with lung injury and thus exemplifying the different metabolic pattern and its associated biochemical alterations exhibited in mild and moderate/severe ARDS. The above pilot study helped in identifying diagnostic signatures based on ARDS substages. But the subsequent biomarker credentials based on severity and progression requires more such follow up and outcome studies with validation in large sample size. The above mentioned limitation is on the pipeline to further establish the role of the proposed biomarker model in the clinical setting.

Conclusion

In conclusion, NMR based metabolomics study was performed to fingerprint the metabolites depicting different stages of ARDS and their impact in the associated biological pathway suggesting their possible role in susceptibility/recuperation. Iterative statistics led to the compilation of 6 key metabolites which could differentiate mild and moderate/severe ARDS, thus arriving at a conclusive predictive biomarker model. The pivotal biological roles of these biomarker candidates investigated through pathway analysis can further establish their contribution to ARDS complex heterogeneity and disease manifested systemic response. The prior research findings are based on pilot study which necessitates streamlining the putative biomarker model in future prospective cohort studies to establish its diagnostic accuracy and potency. Further research into these biomarkers can give better understanding of the pathophysiology, progression and their possible utility and applicability as therapeutic targets. Our study infers NMR based metabolomics as a plausible tool with multidisciplinary applications to provide biomarker model having potent implication in ARDS management with distinct clinical relevance and downstream effect of metabolome in ARDS stages.

Supporting information

S1 Text. (**Figure A**): Representative 800 MHz ^{1}H–^{13}C HSQC spectrum of mBALF collected from ARDS patient depicting diseased lung-specific metabolites. (**Figure B**): Representative 800 MHz ^{1}H–^{1}H TOCSY spectrum of mBALF collected from ARDS patient depicting diseased lung-specific metabolites. (**Figure C**): Data normalization by Pareto scaling and log

transformation. Branched chain amino acids = BCA. (**Figure D**): a) Two-dimensional and b) Three-dimensional score plot of principal component analysis with red color representing Mild ARDS and green as Moderate/ Severe ARDS, c) Two-dimensional and d) Three-dimensional score plot of partial least squares discriminant analysis with red color representing Mild ARDS and green as Moderate/ Severe ARDS e) values of the classification performance assessed by accuracy, R2 and Q2f) third component best classifies the model shown with asterisk. Principal component = PC, partial least squares discriminant analysis = PLS-DA. (**Figure E**): Variables importance in projection (VIP). (**Figure F**): Mean ± standard error of the nine metabolites is shown with respect to the Trimethylsilylpropanoic acid concentration (relative concentration in arbitrary unit). Trimethylsilylpropanoic acid = TSP, arbitrary unit = au, Branched chain amino acids = BCA. (**Figure G**): a) Volcano plot with red dot showing important metabolites. b) Significant values obtained from volcano plot. PRO = Proline, LYS/ARG = Lysine/arginine, TAU = Taurine, THR = Threonine c) statistical tool empirical Bayesian analysis of metabolites to show the discerning markers d) values obtained from empirical Bayesian analysis of metabolites. Fold change = FC, false discovery rate = FDR, empirical Bayesian analysis of metabolites = EBAM. (**Figure H**): a) and c) Random forest classification error with accuracy b) significant metabolites on the basis of mean decrease accuracy and d) Values of mean decrease accuracy. out of bag error = OOB error. (**Table A**): Classification results obtained from discriminant function analysis of 17 metabolites with prediction accuracy of 94.4%. (**Table B**): Significant metabolites selected by T-test with a threshold p value of <0.05 analogous with Variable importance in projection values. (**Table C**): Discriminant function analyses of nine metabolites with 88.9% correct classification. (**Table D**): The classification result of stepwise discriminant function analysis to weed out 5 discriminating markers with 83.3%correct classification. **(Table E)**: Partial least squares discriminant analysis cross validation details of 6 putative biomarkers with values of the classification performance assessed by accuracy, R2 and Q2and third component best classifies the model.
(PDF)

Author Contributions

Conceptualization: Neeraj Sinha, Akhila Viswan, Chandan Singh, Ratan Kumar Rai, Afzal Azim, Arvind Kumar Baronia.

Data curation: Neeraj Sinha, Akhila Viswan, Chandan Singh, Ratan Kumar Rai, Afzal Azim.

Formal analysis: Akhila Viswan.

Investigation: Akhila Viswan, Chandan Singh, Ratan Kumar Rai, Afzal Azim.

Methodology: Neeraj Sinha, Akhila Viswan, Chandan Singh, Ratan Kumar Rai, Afzal Azim.

Project administration: Neeraj Sinha, Afzal Azim, Arvind Kumar Baronia.

Supervision: Neeraj Sinha, Afzal Azim, Arvind Kumar Baronia.

Validation: Akhila Viswan, Chandan Singh, Ratan Kumar Rai, Afzal Azim, Arvind Kumar Baronia.

Visualization: Akhila Viswan, Chandan Singh, Ratan Kumar Rai, Afzal Azim, Arvind Kumar Baronia.

Writing – original draft: Akhila Viswan.

Writing – review & editing: Neeraj Sinha, Akhila Viswan, Afzal Azim.

References

1. Villar J, Blanco J, Añón J, Santos-Bouza A, Blanch L, Ambrós A, et al. (2011) The ALIEN study: incidence and outcome of acute respiratory distress syndrome in the era of lung protective ventilation. Intensive Care Med 37: 1932–1941. https://doi.org/10.1007/s00134-011-2380-4 PMID: 21997128
2. Rubenfeld GD, Caldwell E, Peabody E, Weaver J, Martin DP, Neff M, et al. (2005) Incidence and Outcomes of Acute Lung Injury. N Engl J Med 353: 1685–1693. https://doi.org/10.1056/NEJMoa050333 PMID: 16236739
3. Piantadosi CA, Schwartz DA (2004) The Acute Respiratory Distress Syndrome. Ann Intern Med 141: 460–470. PMID: 15381520
4. The ADTF, Ranieri V, Rubenfeld G, Thompson B, Ferguson N, Caldwell E, et al. (2012) Acute respiratory distress syndrome: The berlin definition. JAMA 307: 2526–2533. https://doi.org/10.1001/jama.2012.5669 PMID: 22797452
5. Ashbaugh D, Boyd Bigelow D, Petty T, Levine B (1967) Acute Respiratory Distress in Adults. The Lancet 290: 319–323.
6. Ware LB, Matthay MA (2000) The Acute Respiratory Distress Syndrome. N Engl J Med 342: 1334–1349. https://doi.org/10.1056/NEJM200005043421806 PMID: 10793167
7. Wheeler AP, Bernard GR (2007) Acute lung injury and the acute respiratory distress syndrome: a clinical review. The Lancet 369: 1553–1564.
8. Briel M, Meade M, Mercat A, Brower R, Talmor D, Walter S, et al. (2010) Higher vs lower positive end-expiratory pressure in patients with acute lung injury and acute respiratory distress syndrome: Systematic review and meta-analysis. JAMA 303: 865–873. https://doi.org/10.1001/jama.2010.218 PMID: 20197533
9. Kopterides P, Siempos II, Armaganidis A. Prone positioning in hypoxemic respiratory failure: Meta-analysis of randomized controlled trials. J Crit Care 24: 89–100. https://doi.org/10.1016/j.jcrc.2007.12.014 PMID: 19272544
10. Serpa Neto A, Cardoso S, Manetta J, Pereira V, Espósito D, Pasqualucci M, et al. (2012) Association between use of lung-protective ventilation with lower tidal volumes and clinical outcomes among patients without acute respiratory distress syndrome: A meta-analysis. JAMA 308: 1651–1659. https://doi.org/10.1001/jama.2012.13730 PMID: 23093163
11. Walkey AJ, Summer R, Ho V, Alkana P (2012) Acute respiratory distress syndrome: epidemiology and management approaches. Clin Epidemiol 4: 159–169. https://doi.org/10.2147/CLEP.S28800 PMID: 22866017
12. Bernard GR, Artigas A, Brigham KL, Carlet J, Falke K, Hudson L, et al. (1994) The American-European Consensus Conference on ARDS. Definitions, mechanisms, relevant outcomes, and clinical trial coordination. Am J Respir Crit Care Med 149: 818–824. https://doi.org/10.1164/ajrccm.149.3.7509706 PMID: 7509706
13. Ferguson ND, Frutos-Vivar F, Esteban A, Fernández-Segoviano P, Aramburu JA, Nájera L, et al. (2005) Acute respiratory distress syndrome: Underrecognition by clinicians and diagnostic accuracy of three clinical definitions. Crit Care Med 33: 2228–2234. PMID: 16215375
14. Taylor MM (2005) ARDS Diagnosis and Management: Implications for the Critical Care Nurse. Dimens Crit Care Nurs 24: 197–207. PMID: 16258347
15. Tagliabue M, Casella TC, Zincone GE, Fumagalli R, Salvini E (1994) CT and Chest Radiography in the Evaluation of Adult Respiratory Distress Syndrome. Acta Radiol 35: 230–234. PMID: 8192958
16. Matthay MA, Zimmerman GA (2005) Acute Lung Injury and the Acute Respiratory Distress Syndrome. Am J Respir Cell Mol Biol 33: 319–327. https://doi.org/10.1165/rcmb.F305 PMID: 16172252
17. Matthay MA, Ware LB, Zimmerman GA (2012) The acute respiratory distress syndrome. J Clin Invest 122: 2731–2740. https://doi.org/10.1172/JCI60331 PMID: 22850883
18. Barnett N, Ware LB (2011) Biomarkers in Acute Lung Injury—Marking Forward Progress. Crit Care Clin 27: 661–683. https://doi.org/10.1016/j.ccc.2011.04.001 PMID: 21742222
19. Cross LJM, Matthay MA. Biomarkers in Acute Lung Injury: Insights into the Pathogenesis of Acute Lung Injury. Crit Care Clin 27: 355–377. https://doi.org/10.1016/j.ccc.2010.12.005 PMID: 21440206
20. Levitt JE, Gould MK, Ware LB, Matthay MA (2009) Analytic Review: The Pathogenetic and Prognostic Value of Biologic Markers in Acute Lung Injury. J Intensiv Care Med 24: 151–167.
21. Cribbs SK, Martin GS (2008) Biomarkers in acute lung injury: are we making progress? Crit Care Med 36: 2457–2459. https://doi.org/10.1097/CCM.0b013e318181052a PMID: 18664801
22. Connelly M, Kevin G, Repine M, John E. (1997) Markers for predicting the development of Acute Respiratory Distress Syndrome. Annu Rev Med 48: 429–445. https://doi.org/10.1146/annurev.med.48.1.429 PMID: 9046974

23. Gebregiworgis T, Powers R (2012) Application of NMR Metabolomics to Search for Human Disease Biomarkers. Comb Chem High Throughput Screening 15: 595–610.
24. Nicholson JK, Lindon JC (2008) Systems biology: Metabonomics. Nature 455: 1054–1056. https://doi.org/10.1038/4551054a PMID: 18948945
25. Meyer NJ (2013) Future clinical applications of genomics for acute respiratory distress syndrome. Lancet Respir Med 1: 793–803. https://doi.org/10.1016/S2213-2600(13)70134-6 PMID: 24461759
26. Alpantaki K, Tsiridis E, Pape H-C, Giannoudis PV (2007) Application of clinical proteomics in diagnosis and management of trauma patients. Injury 38: 263–271. https://doi.org/10.1016/j.injury.2006.11.021 PMID: 17291504
27. Rogers AJ, Matthay MA (2014) Applying metabolomics to uncover novel biology in ARDS. Am J Physiol Lung Cell Mol Physiol 306: L957–L961. https://doi.org/10.1152/ajplung.00376.2013 PMID: 24727586
28. Stringer KA, McKay RT, Karnovsky A, Quémerais B, Lacy P (2016) Metabolomics and Its Application to Acute Lung Diseases. Frontiers in Immunology 7: 44. https://doi.org/10.3389/fimmu.2016.00044 PMID: 26973643
29. Singh C, Rai R, Azim A, Sinha N, Ahmed A, Singh K, et al. (2015) Metabolic profiling of human lung injury by 1H high-resolution nuclear magnetic resonance spectroscopy of blood serum. Metabolomics 11: 166–174.
30. Carpenter CT, Price PV, Christman BW (1998) EXhaled breath condensate isoprostanes are elevated in patients with acute lung injury or ards. Chest 114: 1653–1659. PMID: 9872202
31. Albertine KH, Soulier MF, Wang Z, Ishizaka A, Hashimoto S, Zimmerman GA, et al. Fas and Fas Ligand Are Up-Regulated in Pulmonary Edema Fluid and Lung Tissue of Patients with Acute Lung Injury and the Acute Respiratory Distress Syndrome. Am J Pathol 161: 1783–1796. https://doi.org/10.1016/S0002-9440(10)64455-0 PMID: 12414525
32. Stringer KA, Serkova NJ, Karnovsky A, Guire K, Paine R, Standiford TJ (2010) Metabolic consequences of sepsis-induced acute lung injury revealed by plasma 1H-nuclear magnetic resonance quantitative metabolomics and computational analysis. Am J Physiol Lung Cell Mol Physiol 300: L4–L11. https://doi.org/10.1152/ajplung.00231.2010 PMID: 20889676
33. Howrylak JA, Dolinay T, Lucht L, Wang Z, Christiani DC, Sethi JM, et al. (2009) Discovery of the gene signature for acute lung injury in patients with sepsis. Physiol Genomics 37: 133–139. https://doi.org/10.1152/physiolgenomics.90275.2008 PMID: 19174476
34. McClintock DE, Ware LB, Eisner MD, Wickersham N, Thompson BT, Matthay MA (2007) Higher Urine Nitric Oxide Is Associated with Improved Outcomes in Patients with Acute Lung Injury. Am J Respir Crit Care Med 175: 256–262. https://doi.org/10.1164/rccm.200607-947OC PMID: 17082495
35. Chang DW, Hayashi S, Gharib SA, Vaisar T, King ST, Tsuchiya M, et al. (2008) Proteomic and Computational Analysis of Bronchoalveolar Proteins during the Course of the Acute Respiratory Distress Syndrome. Am J Respir Crit Care Med 178: 701–709. https://doi.org/10.1164/rccm.200712-1895OC PMID: 18658106
36. Rai R, Azim A, Sinha N, Sahoo J, Singh C, Ahmed A, et al. (2013) Metabolic profiling in human lung injuries by high-resolution nuclear magnetic resonance spectroscopy of bronchoalveolar lavage fluid (BALF). Metabolomics 9: 667–676.
37. Serkova NJ, Van Rheen Z, Tobias M, Pitzer JE, Wilkinson JE, Stringer KA (2008) Utility of magnetic resonance imaging and nuclear magnetic resonance-based metabolomics for quantification of inflammatory lung injury. Am J Physiol Lung Cell Mol Physiol 295: L152–L161. https://doi.org/10.1152/ajplung.00515.2007 PMID: 18441091
38. Ciaramelli C, Fumagalli M, Viglio S, Bardoni AM, Piloni D, Meloni F, et al. (2017) 1H NMR To Evaluate the Metabolome of Bronchoalveolar Lavage Fluid (BALf) in Bronchiolitis Obliterans Syndrome (BOS): Toward the Development of a New Approach for Biomarker Identification. J Proteome Res 16: 1669–1682. https://doi.org/10.1021/acs.jproteome.6b01038 PMID: 28245130
39. Hu JZ, Rommereim DN, Minard KR, Woodstock A, Harrer BJ, Wind RA, et al. (2008) Metabolomics in Lung Inflammation:A High-Resolution 1H NMR Study of Mice Exposedto Silica Dust. Toxicol Mech Methods 18: 385–398. https://doi.org/10.1080/15376510701611032 PMID: 20020862
40. Wolak JE, Esther CR, O'Connell TM (2009) Metabolomic analysis of bronchoalveolar lavage fluid from cystic fibrosis patients. Biomarkers 14: https://doi.org/10.1080/13547500802688194 PMID: 19283525
41. Airoldi C, Ciaramelli C, Fumagalli M, Magni S, Viglio S, Piloni D, et al. (2016) Optimizing a protocol for the NMR-based metabolic profiling of BALf in bronchiolitis obliterans syndrome. Eur Respir J 48.
42. Beckonert O, Keun HC, Ebbels TM, Bundy J, Holmes E, Lindon JC, et al. (2007) Metabolic profiling, metabolomic and metabonomic procedures for NMR spectroscopy of urine, plasma, serum and tissue extracts. Nat Protoc 2: 2692–2703. https://doi.org/10.1038/nprot.2007.376 PMID: 18007604

43. Lindon JC, Holmes E, Nicholson JK (2004) Metabonomics and its role in drug development and disease diagnosis. Expert Rev Mol Diagn 4: 189–199. https://doi.org/10.1586/14737159.4.2.189 PMID: 14995905

44. Nicholson JK, Holmes E, Kinross JM, Darzi AW, Takats Z, Lindon JC (2012) Metabolic phenotyping in clinical and surgical environments. Nature 491: 384–392. https://doi.org/10.1038/nature11708 PMID: 23151581

45. Evans CR, Karnovsky A, Kovach MA, Standiford TJ, Burant CF, Stringer KA (2014) Untargeted LC—MS Metabolomics of Bronchoalveolar Lavage Fluid Differentiates Acute Respiratory Distress Syndrome from Health. J Proteome Res 13: 640–649. https://doi.org/10.1021/pr4007624 PMID: 24289193

46. Singh C, Rai R, Azim A, Sinha N, Baronia A (2013) Mini-bronchoalveolar lavage fluid can be used for biomarker identification in patients with lung injury by employing 1H NMR spectroscopy. Crit Care 17: 430. https://doi.org/10.1186/cc12607 PMID: 23635380

47. Viswan A, Sharma RK, Azim A, Sinha N (2016) NMR-Based Metabolic Snapshot from Minibronchoalveolar Lavage Fluid: An Approach To Unfold Human Respiratory Metabolomics. J Proteome Res 15: 302–310. https://doi.org/10.1021/acs.jproteome.5b00919 PMID: 26587756

48. Singh C, Rai RK, Azim A, Sinha N, Baronia AK (2014) Search for biomarkers in critically ill patients: a new approach based on nuclear magnetic resonance spectroscopy of mini-bronchoalveolar lavage fluid. Critical Care 18: 594. https://doi.org/10.1186/s13054-014-0594-x PMID: 25672613

49. Villar J, Blanco J, Kacmarek RM (2016) Current incidence and outcome of the acute respiratory distress syndrome. Current Opinion in Critical Care 22: 1–6. https://doi.org/10.1097/MCC.0000000000000266 PMID: 26645551

50. Kapil A, Khilnani G, Sharma S, Sood S, Arafath TK, Hadda V (2011) Comparison of bronchoscopic and non-bronchoscopic techniques for diagnosis of ventilator associated pneumonia. Indian J Crit Care Med 15: 16–23. https://doi.org/10.4103/0972-5229.78218 PMID: 21633541

51. Xia J, Psychogios N, Young N, Wishart DS (2009) MetaboAnalyst: a web server for metabolomic data analysis and interpretation. Nucleic Acids Res 37: W652–W660. https://doi.org/10.1093/nar/gkp356 PMID: 19429898

52. Andersen LW, Mackenhauer J, Roberts JC, Berg KM, Cocchi MN, Donnino MW. Etiology and Therapeutic Approach to Elevated Lactate Levels. Mayo Clin Proc 88: 1127–1140. https://doi.org/10.1016/j.mayocp.2013.06.012 PMID: 24079682

53. Li B, Tang J, Yang Q, Cui X, Li S, Chen S, et al. (2016) Performance Evaluation and Online Realization of Data-driven Normalization Methods Used in LC/MS based Untargeted Metabolomics Analysis. Sci Rep 6: 38881. https://doi.org/10.1038/srep38881 PMID: 27958387

54. van den Berg RA, Hoefsloot HC, Westerhuis JA, Smilde AK, van der Werf MJ (2006) Centering, scaling, and transformations: improving the biological information content of metabolomics data. BMC Genomics 7: 142. https://doi.org/10.1186/1471-2164-7-142 PMID: 16762068

55. Ivosev G, Burton L, Bonner R (2008) Dimensionality Reduction and Visualization in Principal Component Analysis. Anal Chem 80: 4933–4944. https://doi.org/10.1021/ac800110w PMID: 18537272

56. Parsons HM, Ludwig C, Günther UL, Viant MR (2007) Improved classification accuracy in 1- and 2-dimensional NMR metabolomics data using the variance stabilising generalised logarithm transformation. BMC Bioinformatics 8: 234. https://doi.org/10.1186/1471-2105-8-234 PMID: 17605789

57. Lenz A, Jorens P, Meyer B, De Backer W, Van Overveld F, Bossaert L, et al. (1999) Oxidatively modified proteins in bronchoalveolar lavage fluid of patients with ARDS and patients at-risk for ARDS. Eur Respir J 13: 169–174. PMID: 10836343

58. Shen L, Luo Z-Q, Han J-Z, Yue S-J, Qin X-Q, Li C, et al. (2007) Experimental study of acute lung injury induced by glutamate in vivo. Zhong Nan Da Xue Xue Bao Yi Xue Ban 32: 78–81. PMID: 17344592

59. Wang M, Luo Z, Liu S, Li L, Deng X, Huang F, et al. (2009) Glutamate Mediates Hyperoxia-Induced Newborn Rat Lung Injury through N-Methyl-D-Aspartate Receptors. Am J Respir Cell Mol Biol 40: 260–267. https://doi.org/10.1165/rcmb.2008-0135OC PMID: 18757305

60. Said SI, Berisha HI, Pakbaz H (1996) Excitotoxicity in the lung: N-methyl-D-aspartate-induced, nitric oxide-dependent, pulmonary edema is attenuated by vasoactive intestinal peptide and by inhibitors of poly(ADP-ribose) polymerase. Proc Natl Acad Sci U S A 93: 4688–4692. PMID: 8643465

61. Abdih H, Kelly CJ, Bouchier-Hayes D, Barry M, Kearns S (2000) Taurine prevents interleukin-2-induced acute lung injury in rats. Eur Surg Res 32: 347–352. PMID: 11182618

62. Zhou M, Martindale RG (2007) Arginine in the Critical Care Setting. J Nutr 137: 1687S–1692S. PMID: 17513448

63. Mitani Y, Maruyama K, Sakurai M (1997) Prolonged Administration of l-Arginine Ameliorates Chronic Pulmonary Hypertension and Pulmonary Vascular Remodeling in Rats. Circulation 96: 689–697. PMID: 9244244

64. Dweik RA (2007) The lung in the balance: arginine, methylated arginines, and nitric oxide. Am J Physiol Lung Cell Mol Physiol 292: L15–L17. https://doi.org/10.1152/ajplung.00322.2006 PMID: 16980373

65. Carter BW, Chicoine LG, Nelin LD (2004) l-Lysine Decreases Nitric Oxide Production and Increases Vascular Resistance in Lungs Isolated from Lipopolysaccharide-Treated Neonatal Pigs. Pediatr Res 55: 979–987. https://doi.org/10.1203/01.pdr.0000127722.55965.b3 PMID: 15155866

66. Chiarla C, Giovannini I, Siegel JH, Boldrini G, Castagneto M (2000) The Relationship between Plasma Taurine and Other Amino Acid Levels in Human Sepsis. J Nutr 130: 2222–2227. PMID: 10958816

67. Li P, Yin Y-L, Li D, Woo Kim S, Wu G (2007) Amino acids and immune function. Br J Nutr 98: 237–252. https://doi.org/10.1017/S000711450769936X PMID: 17403271

68. Maarsingh H, Dekkers BGJ, Zuidhof AB, Bos IST, Menzen MH, Klein T, et al. (2011) Increased arginase activity contributes to airway remodelling in chronic allergic asthma. Eur Respir J 38: 318–328. https://doi.org/10.1183/09031936.00057710 PMID: 21310883

69. Phang JM, Pandhare J, Liu Y (2008) The Metabolism of Proline as Microenvironmental Stress Substrate. J Nutr 138: 2008S–2015S. PMID: 18806116
